# Surface‐Assisted Synthesis of N*‐*Containing *π*‐Conjugated Polymers

**DOI:** 10.1002/advs.202200407

**Published:** 2022-05-22

**Authors:** Ana Sánchez‐Grande, José I. Urgel, Inés García‐Benito, José Santos, Kalyan Biswas, Koen Lauwaet, José M. Gallego, Johanna Rosen, Rodolfo Miranda, Jonas Björk, Nazario Martín, David Écija

**Affiliations:** ^1^ IMDEA Nanoscience C/ Faraday 9, Campus de Cantoblanco Madrid 28049 Spain; ^2^ Departamento de Química Orgánica. Facultad de Ciencias Químicas Universidad Complutense Madrid 28040 Spain; ^3^ Instituto de Ciencia de Materiales de Madrid CSIC Cantoblanco Madrid 28049 Spain; ^4^ Department of Physics Chemistry and Biology IFM Linköping University Linköping 58183 Sweden; ^5^ Departamento de Física de la Materia Condensada Universidad Autónoma de Madrid Madrid 28049 Spain

**Keywords:** conjugated polymers; N*‐*heteroacenes, scanning probe microscopies, on‐surface synthesis

## Abstract

On‐surface synthesis has recently emerged as a powerful strategy to design conjugated polymers previously precluded in conventional solution chemistry. Here, an N‐containing pentacene‐based precursor (tetraazapentacene) is ex‐professo synthesized endowed with terminal dibromomethylene (:CBr_2_) groups to steer homocoupling via dehalogenation on metallic supports. Combined scanning probe microscopy investigations complemented by theoretical calculations reveal how the substrate selection drives different reaction mechanisms. On Ag(111) the dissociation of bromine atoms at room temperature triggers the homocoupling of tetraazapentacene units together with the binding of silver adatoms to the nitrogen atoms of the monomers giving rise to a N‐containing conjugated coordination polymer (**P1**). Subsequently, **P1** undergoes ladderization at 200 °C, affording a pyrrolopyrrole‐bridged conjugated polymer (**P2**). On Au(111) the formation of the intermediate polymer **P1** is not observed and, instead, after annealing at 100 °C, the conjugated ladder polymer **P2** is obtained, revealing the crucial role of metal adatoms on Ag(111) as compared to Au(111). Finally, on Ag(100) the loss of :CBr2 groups affords the formation of tetraazapentacene monomers, which coexist with polymer **P1**. Our results contribute to introduce protocols for the synthesis of N‐containing conjugated polymers, illustrating the selective role of the metallic support in the underlying reaction mechanisms.

## Graphical abstract

A tetraazapentacene precursor endowed with =CBr_2_ groups is synthesized to steer unprecedented on‐surface polymerizations. On Ag(111) an intermediate conjugated coordination polymer (**P1**) is detected, which evolves towards a conjugated ladder polymer **P2** upon annealing. Counterintuitively, on Au(111) **P2** is found at a much lower annealing temperature, without the observation of **P1**, thus highlighting the decisive role of the substrate.

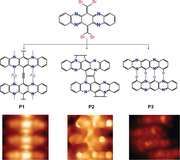

## 1 Introduction

During the last decade, on‐surface synthesis has emerged as a powerful and successful strategy toward the synthesis of carbon‐based nanomaterials such as nanographenes (NGs)^[^ ^1^, ^2^, ^3^ ^]^ or 1D^[^ ^4^, ^5^, ^6^ ^]^ and 2D *π*‐conjugated polymers^[^ ^7^, ^8^ ^]^ that are frequently challenging to obtain in solution chemistry due to their high reactivity and/or low solubility. For instance, the recent synthesis of several members of the acene family^[^ ^9^, ^10^, ^11^, ^12^, ^13^ ^]^ or acene‐based 1D polymers^[^ ^14^, ^15^ ^]^ supported on metal substrates has supposed a great progress in this field in view of their unique electronic, magnetic, and optical properties, with potential applications in organic (opto)electronics and spintronics.^[^ ^16^, ^17^ ^]^ Among the existing strategies to tune their remarkable properties, heteroatom substitution allows to tailor the topology of the *π*‐conjugation by replacing CH groups from the carbon skeleton by N, B, P, or S atoms.^[^ ^18^, ^19^, ^20^ ^]^ In particular, in the case of N*‐*containing acenes, so called azaacenes or N*‐*heteroacenes, it has been demonstrated that N*‐*doping is an efficient strategy to engineer their electronic bandgap and/or to induce an open‐shell character in their ground state.^[^ ^21^, ^22^, ^23^ ^]^

Contemporarily to the progress made in the synthesis of *π*‐conjugated systems on surfaces, the fabrication of low dimensional coordinative metal–organic nanoarchitectures based on metal ions and *π*‐conjugated ligands has been extensively explored.^[^ ^24^, ^25^, ^26^, ^27^, ^28^ ^]^ Herein, the on‐surface chemistry toolbox of *π*–d systems, achieved by direct deposition of molecular ligands that coordinate with substrate adatoms or by codeposition of different metals and ligands, leading to the hybridization of the d‐orbitals of transition metals and frontier orbitals of conjugated ligands, has been extensively investigated.

Most of the works related to on‐surface synthesis have been realized on noble metal substrates in order to steer desired chemical reactions by exploiting the catalytic role of the metallic support. Hereby, the selection of the substrate is crucial for the evolution of the reaction and, actually, the same precursor can give rise to different reaction pathways and products on distinct metals. Therefore, the reaction mechanisms involved in an on‐surface reaction depends on the choice of both the surface and the organic precursor.^[^ ^29^, ^30^, ^31^, ^32^ ^]^

In this work, we present a comprehensive study based on scanning tunneling microscopy (STM), scanning tunneling spectroscopy (STS), and noncontact atomic force microscopy (nc‐AFM), complemented by density functional theory (DFT), to unveil the role of the metal substrate in the formation of new conjugated polymers, revealing substrate‐selective synthetic pathways toward novel reaction products. With this aim, we employ a N*‐*containing pentacene‐based (tetraazapentacene) precursor, termed 4Br4AzaPn, which is endowed with dibromomethylene groups (:CBr_2_) to steer dehalogenation and homocoupling at a first stage, and ladderization at a second one,^[^ ^14^, ^33^, ^34^ ^]^ upon thermal annealing on gold or silver substrates.

**Scheme 1:**
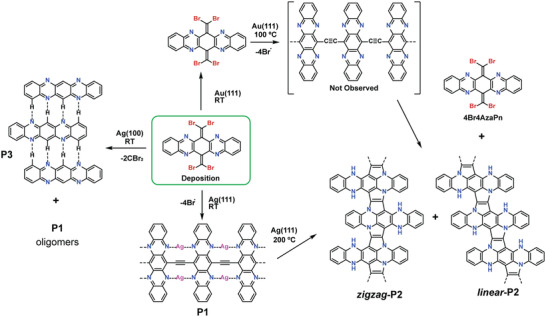
Reaction pathways of **4Br4AzaPn** on Au(111), Ag(111), and Ag(100).

The deposition of **4Br4AzaPn** on Ag(111) at room temperature (RT) gives rise to the formation of a 1D polymer constituted by central ethynylene bridges and peripheral N*‐*Ag‐N links (product **P1**), thus affording both *π*–*π* and *π*–d conjugation pathways. Subsequent annealing at 200 °C results in an unprecedented pyrrolopyrrole N*‐*containing conjugated ladder polymer (**P2**) through a novel reaction pathway (cf. **Scheme** **1**).

Notably, following the same strategy on Au(111), we only observe intact precursors at RT, while once thermal annealing surpasses 100 °C, the formation of the conjugated ladder polymer **P2** emerges, without the detection of any intermediate ethynylene‐bridged polymer, which reveals the pivotal role of the metal substrate (cf. Scheme 1). Our results indicate that on Ag(111) the presence of silver‐directed metal–organic bonds stabilizes polymer **P1**, thus implying a higher annealing temperature to steer ladderization, as compared to Au(111).

Finally, in order to further corroborate the role of the Ag surface in the formation of **P1**, **4Br4AzaPn** was deposited onto a pristine Ag(100) substrate at RT, giving rise to the formation of oligomers of **P1** and tetraazapentacene monomers (**P3**) after the cleavage of both :CBr_2_ groups (cf. Scheme 1).

Our study introduces the crucial role of metal supports to specifically direct both reaction mechanisms and products. We envision that these results will contribute to develop the on‐surface synthesis field, while expanding the chemical design of N*‐*containing conjugated nanomaterials.

## 2 Results

### 2.1 Ag(111) Surface

**Figure 1:**
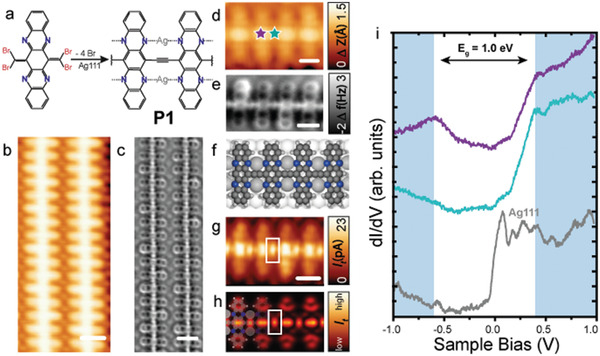
Structural and electronic characterization of **P1** on Ag(111). a) Chemical scheme illustrating the on‐surface synthesis of **P1**. b) Overview STM image after depositing a submonalayer coverage of **4Br4AzaPn** on Ag(111) at RT. *V*_b_ = 200 mV, *I*_t_ = 10 pA, scale bar = 1 nm. c) nc‐AFM image of (b). *V*_b_ = 3 mV, scale bar = 1 nm. d) HR‐STM image of **P1**. *V*_b_ = −1 V, *I*_t_ = 350 pA, and scale bar = 6 Å. e) Nc‐AFM image of **P1** revealing a bright protrusion at the middle of the ethynylene bridge, which is attributed to a triple bond. *V*_b_ = 3 mV, scale bar = 6 Å. f) DFT model of the ethynylene‐bridged N*‐*containing pentacene polymer with two Ag adatoms bonded to the nitrogen atoms per unit cell (dark, blue, white, and light grey balls correspond to carbon, nitrogen, silver surface atoms, and silver adatoms, respectively). g) Constant‐height STM image of **P1** showing an increased density of unoccupied states located perpendicularly to the triple bond highlighted by a white square. *V*_b_ = 3 mV, scale bar = 6 Å. h) Simulated constant‐height STM image of **P1**. i) Scanning tunneling spectra acquired on the positions depicted in (d) by the purple and cyan stars and reference spectrum taken on the bare Ag(111) surface (grey line).

Sublimation of **4Br4AzaPn** (for synthetic details see the Supporting Information) onto a pristine Ag(111) held at RT leads to the spontaneous formation of 1D linear polymers, as illustrated in the chemical sketch and the STM and nc‐AFM images shown in **Figure** **1**a–c, respectively. The representative high‐resolution STM image in Figure 1d reveals that tetraazapentacene units are linearly connected with each other through the central rings. Constant‐height frequency‐shift nc‐AFM measurements acquired with a CO‐functionalized tip allows to discern the tetraazapentacene backbone and illustrates the ethynylene‐like nature of the connection between the N*‐*containing pentacene units, which is expressed as an enhanced contrast at their central positions, characteristic of a triple bond,^[^ ^35^ ^]^ as displayed in Figure 1e.

In addition, Laplace‐filtered frequency‐shift images show faint features of an increased frequency shift tentatively assigned at this point to Ag adatoms,^[^ ^36^ ^]^ being chelated by the nitrogen atoms of adjacent tetraazapentacene units (pointed with white arrows in Figure S1 in the Supporting Information), thus establishing two‐fold N*‐*Ag‐N bonds (cf. scheme in Figure 1a). In order to corroborate such hypothesis, DFT calculations of **P1** (cf. model in Figure 1f), with and without Ag adatoms coordinated to two N atoms of adjacent monomeric units, were performed. In the absence of adatoms, the ethynylene bridge interacts strongly with the surface and the simulated STM images do not resemble the experimental ones (cf. Figure S2, Supporting Information). Particularly, the inequivalent positions of the ethynylene bridges with respect to the Ag(111) surface would show different contrast in different parts of the polymer in the STM. When coordinated to Ag adatoms, the polymer is lifted away from the surface such that its units become more structurally equivalent (cf. Figure S2, Supporting Information). This findings are also reflected in the STM images, in which the constant‐height STM image at low bias voltage (cf. Figure 1g) shows an excellent agreement with the simulated constant‐height image around the Fermi level (cf. Figure 1h), whereby an increase in the electronic density is observed surrounding the ethynylene bridge (highlighted with a white square). It is also worth mentioning the exceptional agreement between the experimental STM image at −1.0 V (cf. Figure 1d) and the simulated constant‐current image in Figure S2f in the Supporting Information.

Next, we have investigated the electronic properties of **P1** by means of STS. The differential conductance d*I*/d*V* spectra shown in Figure 1i suggest a low bandgap of 1.0 eV, in which we propose that the N*‐*Ag‐N coordination is favoring the electronic delocalization. Altogether, **P1** can thus be considered as a 1D coordination conjugated polymer (CCP), displaying both *π*–*π* and *π*–d conjugation pathways and featuring a low bandgap.

Further annealing at 150 °C does not lead to any significant modification in the polymer structure of **P1** on Ag(111) (cf. Figure S3, Supporting Information), confirming the temperature‐stability of such polymer. However, thermal activation at 200 °C promotes the cleavage of the N*‐*Ag‐N bonds and the formation of a *π*‐conjugated ladder polymer (**P2**), via an unprecedented ladderization reaction, as shown in Figure S3 (Supporting Information) and described in detail below.

### 2.2 Au(111) Surface

**Figure 2:**
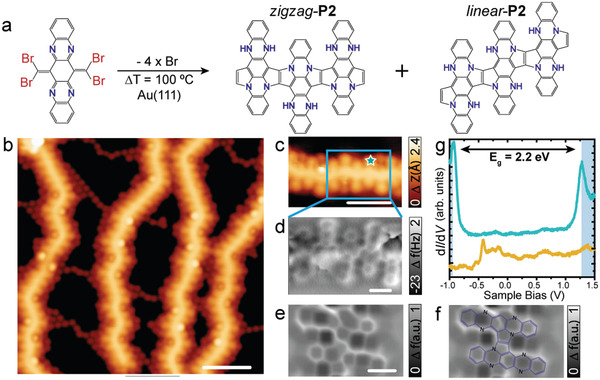
Structural and electronic characterization of **P2** on Au(111). a) Chemical scheme illustrating the on‐surface synthesis of **P2**. b) Overview STM image of the deposition of a submonolayer coverage of **4Br4AzaPn** precursor on Au(111) after annealing at 100 °C. *V*_b_ = 1 V, *I*_t_ = 25 pA, scale bar = 3 nm. c) HR‐STM image of **P2**. *V*_b_ = 20 mV, *I*_t_ = 5 pA, scale bar = 2 nm. d) nc‐AFM image of **P2** showing the non‐planar twisted geometry. e) Laplace filtered nc‐AFM image confirming the formation of the pyrrolopyrrole bridge. f) Superposition of chemical scheme on (e). d–f) *V*_b_ = 5 mV, scale bar = 5 Å. g) Scanning tunneling spectra acquired on the position depicted in (c) and on Au(111).

To investigate the role of the Ag(111) surface and the silver adatoms, we deposited the precursor on two additional substrates, namely Au(111) and Ag(100), and inspected the reaction products. First, **4Br4AzaPn** was sublimed on top of a pristine Au(111) surface held at RT. After sublimation, we observe the intact precursor on the surface, as shown in Figure S4 in the Supporting Information. However, thermal activation at 100 °C induces dissociation of bromine atoms from the :CBr_2_ moieties and the formation of polymer **P2** (cf. **Figure** **2**a–d), which coexists with minority intact **4Br4AzaPn** species (cf. Figure S5, Supporting Information). Importantly, the intermediate ethynylene‐bridged polymer is not detected (cf. Scheme 1).

The chemical nature of **P2** was elucidated by nc‐AFM measurements, revealing the non‐planarity of the polymer (cf. Figure 2d). Interestingly, Figure 2e displays a Laplace‐filtered frequency‐shift image where the formation of the pyrrolopyrrole bridge connecting the tetraazapentacene units is discerned (cf. Figure 2f with the chemical model overlayed). The ladderization toward the formation of the pyrrolopyrrole bridge can give rise to either linear‐ or zigzag‐configurations, as shown in the scheme of Figure 2a, whose junctions afford straight or curved segments. Notably, each tetraazapentacene unit presents a twisted geometry, featuring one side up and the other one down.^[^ ^37^ ^]^ We tentatively attribute such adsorption geometry to the hydrogenation of the nitrogen atoms from pyrazine rings (cf. scheme in Figure 2a), which gives rise to steric hindrance between adjacent tetraazapentacene units, as previously observed for other N*‐*containing acenes^[^ ^23^ ^]^ and as illustrated in Figure S6 (Supporting Information) by DFT calculations of freestanding **P2** with and without extra hydrogenation. Notably, such extra hydrogenation is taking place in the majority of the units, with some exceptions where the tetraazapentacene monomers adopt a more planar configuration allowing to discern the nature of the pyrrolopyrrole bridge (like the example shown in Figure 2e,f).

Next, we have investigated the electronic properties of **P2** via STS. The differential conductance spectrum in Figure 2g suggests a bandgap of 2.2 eV, revealing the onsets of the valence band (VB) and the conduction band (CB) at −0.9 and 1.25 eV, respectively.

### 2.3 Ag(100) Surface

**Figure 3:**
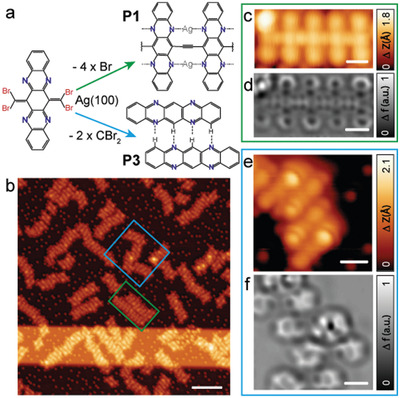
Structural characterization of **P1** and **P3** on Ag(100). a) Chemical scheme of the on‐surface synthesis of **P1** and **P3** on Ag(100). b) Overview STM image of the deposition a submonolayer coverage of **4Br4AzaPn** precursor on Ag(100) revealing the formation of **P1** (green square) and **P3** (blue square). *V*_b_ = 20 mV, *I*_t_ = 5 pA, scale bar = 5 nm. c) HR‐STM image of **P1**. *V*_b_ = 20 mV, *I*_t_ = 5 pA, scale bar = 6 Å. d) Laplace filtered nc‐AFM image of **P1**. *V*_b_ = 3 mV, scale bar = 6 Å. e) HR‐STM image of **P3**. *V*_b_ = 20 mV, *I*_t_ = 5 pA, scale bar = 8 Å. f) Laplace filtered nc‐AFM image of P3. *V*_b_ = 3 mV, scale bar = 5 Å.

Finally, **4Br4AzaPn** was sublimated on Ag(100) with the substrate held at RT, which results in the formation of two different products (cf. **Figure** **3**a,b). On one hand, we detect the synthesis of oligomers of **P1** (cf. Figure 3c,d; Figure SI7, Supporting Information), which are identical to the one found on Ag(111). The length of the 1D chains is shorter compared to Ag(111), with only a maximum number of 5 tetraazapentacene units per chain. On the other hand, the on‐surface synthesis of 5,7,12,14‐tetraazapentacene **P3** is observed (cf. Figure 3e,f; Figure SI7, Supporting Information), due to the high reactivity of the Ag(100) surface, which induces the loss of the :CBr_2_ groups and precludes diffusion, thus not affording homocoupling. DFT calculations of **P3** were performed considering both the scenarios where the :CBr_2_ groups are either removed or replaced by hydrogen atoms (Figures S8–S10, Supporting Information), for which the STM simulations of **P3** with additional hydrogen was found to have the best resemblance of the experimental STM images (cf. Figure S11, Supporting Information). **P3** monomers are highly curved with the central part pushed down to the surface, as previously encountered for other acenes. The self‐assembly of the monomers is promoted by intermolecular N···H interactions between adjacent molecules.^[^ ^38^ ^]^

### 2.4 Reaction Mechanisms

Considering the different behaviors on Ag(111) and Au(111) it is of interest to understand how the reaction mechanisms differ between the surfaces. In particular, getting knowledge about how important the Ag adatoms are for the formation of ethynylene‐bridged polymer **P1** on Ag(111). To understand the reaction mechanisms toward the synthesis of **P1** and **P2** and the crucial role of the surface, we have performed DFT calculations of the reaction pathways on Ag(111) and Au(111).

**Figure 4:**
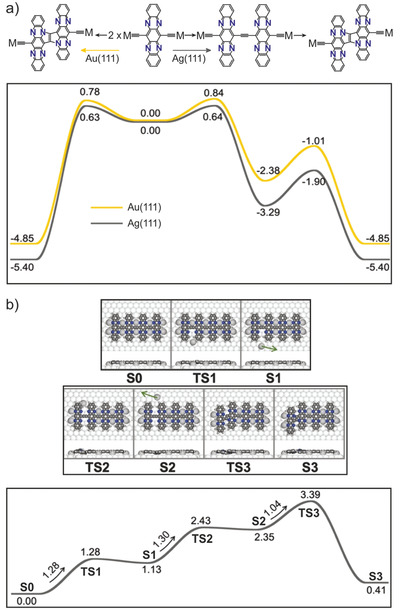
Reaction mechanisms on Ag(111) and Au(111). a) The coupling mechanisms between two dehalogenated monomers on Ag(111) and Au(111), comparing the direct formation of a pyrrolopyrrole connection with the stepwise formation of the pyrrolopyrrole via the ethynylene intermediate. Effective barriers for the different processes are shown and the details of the reactions are given in the Supporting Information. b) The reaction mechanism on Ag(111) of removing adatoms (S0 to S2) and the concomitant ladderization of an ethynylene bridge into a pyrrolopyrrole group (S2 to S3), showing local minima (S0‐S3) and transition states (TS1‐TS3) of the pathway and the associated energy profile. In both (a) and (b) energies are given in units of eV.

One hypothesis is that the different reaction products are a result of different activation energies for making ethynylene and pyrrolopyrrole links on the two surfaces. In **Figure** **4**a, the rate‐limiting steps for making the connections, as well as ladderization of ethynylene into pyrrolopyrrole between two dehalogenated molecular building blocks are compared (the adsorption configurations and complete pathways are shown in in Figures S12–S24 in the Supporting Information). On Ag(111), the activation energy for making the ethynylene bridge is smaller than for the ladderization of ethynylene into pyrrolopyrrole. However, the activation energy to directly form the pyrrolopyrrole is essentially the same as for the initial ethynylene coupling. In other words, from these results one would not expect the formation of exclusively ethynylene connections on the Ag(111) surface. For Au(111) the energy landscape is slightly higher, but the overall picture is the same, and we can expect the direct formation of pyrrolopyrrole as well through the ethynylene intermediate. We also performed calculations comparing the ethynylene to pyrrolopyrrole ladderization of a four‐unit oligomer on the two surfaces, as well as in gas phase. Both surfaces lower the activation energy compared to gas phase; 1.25 and 1.11 eV on Ag(111) and Au(111), respectively, and 1.89 eV in gas phase (cf. Figures S16 and S17, Supporting Information). Again, such activation energies explain why the ethynylene chains cannot be stabilized on Au(111), but they are not consistent with the high temperature needed for the observed transformation on Ag(111). From these results it seems likely that the Ag adatoms, incorporated into the linear chains on Ag(111), must also play a role in the reaction mechanism, hindering the ladderization of ethynylene into pyrrolopyrrole.

In Figure 4b we show a complete reaction step to transform a ethynylene bridge into pyrrolopyrrole on Ag(111), including the removal of the two adatoms from the nitrogen atoms involved in the reaction. The activation energy for the ethynylene to pyrrolopyrrole transformation (**S2** to **S3**) of 1.04 eV is lower than without adatoms (1.25 eV). However, removing the adatoms, necessary to enable the reaction, is highly endothermic, providing a plausible explanation why the ethynylene polymer is stabilized on Ag(111): the temperature needs to be increased such that the entropy of the adatom is sufficient to make coordination to the polymer less favorable compared to diffusing over the surface. Including the entropy of Ag adatoms would in fact have a two‐fold impact, both making the reaction more kinetically accessible and thermodynamically favorable. If considering the free energy of the reaction, for each step where an adatom is removed we would need to add a term − *T*Δ*S*_Ag_, where Δ*S*_Ag_ is the entropy difference of a free Ag adatom and an Ag atom coordinated to the polymer., i.e., the term needs to be added for states **S1** and **TS2** which have one Ag atom less than the initial state **S0**, while for states **S2**, **TS3**, and **S3** there are two adatoms less than **S0**, and − 2*T*Δ*S*_Ag_ has to be added to account for the entropy of the adatoms. The effect of including the entropy is demonstrated in the Figures S25–S27 (Supporting Information), where the entropy of adatoms evaluated using a complete potential energy sampling^[^ ^39^ ^]^ has been included in the energy profile. By doing so, the overall reaction becomes thermodynamically favorable. While estimating the full free energy pathway is not computationally feasible, the results give a qualitative understanding why a relatively high energy is required for the ladderization on Ag(111).

## 3 Conclusions

We have carried out a comprehensive study toward the synthesis of N*‐*containing conjugated polymers by the reaction of a **4Br4AzaPn** precursor sublimed on distinct noble metal supports, namely Ag(111), Au(111) and Ag(100), revealing the influence of the surface in directing the reaction pathways and the obtained products.

On Ag(111), we report the on‐surface synthesis of a CCP based on *π*‐conjugated and N*‐*Ag‐N coordinated tetraazapentacene units (**P1**). Subsequent annealing to 200 °C reveals the formation of an unprecedented conjugated ladder polymer (**P2**), with tetraazapentacene units fused to pyrrolopyrrole bridges.

Conversely and counterintuitively, on Au(111) the reaction of the precursor gives rise to the pyrrolopyrrole polymer already at 100 °C, half the temperature required for the Ag(111) case and without the intermediate **P1** being detected. Such different behavior between Ag(111) and Au(111) is attributed to the role of silver adatoms and their affinity to the tetraazapentacene moieties.

Finally, on Ag(100) the influence of a higher interaction of the **4Br4AzaPn** precursor with the substrate is manifested and the formation of both isolated **P3** monomers and **P1** polymers is observed.

Our study opens new avenues for engineering unique conjugated polymers on distinct metallic supports, while shedding light into the critical role of specific metallic surfaces in driving reaction pathways. Altogether, our findings will contribute to the development of the fields of on‐surface synthesis and polymer materials science in a controlled manner.

## 4 Experimental Section

### Experimental Methods

Experiments were performed in a custom‐designed ultrahigh vacuum system (base pressure below 4 × 10^−10^ mbar) hosting a commercial low‐temperature microscope with STM/AFM capabilities from ScientaOmicron and located at IMDEA Nanoscience (Madrid, Spain).

The metal substrates were prepared by repeated cycles of Ar^+^ sputtering (*E* = 1 keV) and subsequent annealing to 740 K for 10 min. All STM images shown were taken in constant current mode, unless otherwise noted, with electrochemically etched tungsten tips, at a sample temperature of 4.3 K (LakeShore). Scanning parameters are specified in each figure caption. Molecular precursor **4Br4AzaPn** was thermally deposited (Kentax TCE‐BSC) onto the desired clean substate held at RT with a typical deposition rate of 0.1 Å min^−1^ (sublimation temperature of 165 °C), controlled by a quartz micro balance (LewVac). After deposition of **4Br4AzaPn**, the sample was either inspected by scanning probe microscopy or postannealed at indicated temperatures.

Noncontact AFM measurements were performed with a tungsten tip attached to a Qplus tuning fork sensor (ScientaOmicron).^[^ ^40^ ^]^ The tip was a posteriori functionalized by a controlled adsorption of a single CO molecule at the tip apex from a previously CO‐dosed surface. The functionalized tip enables the imaging of the intramolecular structure of organic molecules.^[^ ^41^ ^]^ The sensor was driven at its resonance frequency (≈26 kHz for Qplus) with a constant amplitude of ≈60 pm. The shift in the resonance frequency of the sensor (with the attached CO‐functionalized tip) was recorded in a constant‐height mode (ScientaOmicron Matrix electronics and MFLI PLL by Zurich Instruments for ScienaOmicron). The STM and nc‐AFM images were analyzed using WSxM.^[^ ^42^ ^]^

### Computational Details

Periodic density functional theory calculations were performed with the VASP code,^[^ ^43^ ^]^ using the projector‐augmented wave method^[^ ^44^ ^]^ to describe ion–core interaction, together with a plane wave basis expanded to a kinetic energy cutoff of 400 eV. Exchange‐correlation were described by the van der Waals density functional (vdW‐DF),^[^ ^45^, ^46^ ^]^ in the recent form by Hamada,^[^ ^47^ ^]^ denoted by rev‐vdW‐DF2, which has shown to accurately describe molecular adsorption on gold and silver surfaces.^[^ ^48^, ^49^ ^]^ The Ag(111) and Au(111) surfaces were represented by four layered slabs. For the calculations of the monomer adsorption as well as the coupling between two monomers a *p*(8 × 8) surface unit cell together with a 4 × 4 *k*‐point sampling for Ag(111) and a 8 × 8 *k*‐point sampling for Au(111). The relatively large *k*‐point sampling was needed due to the strong interaction between dehalogenated monomer and the surfaces. For the calculations of oligomers with four and six monomers units, we a *p*(8 × 13) and a (8 × 18) unit cell, respectively. Γ‐point only k‐point sampling was sufficient in these cases. The Ag(100) surfaces were also represented by four layered slabs. For isolated molecules, a *p*(8 × 6) unit cell together with a 3 × 3 *k*‐point was used, while for the **P3** polymers we used a *p*(8 × 4) surface unit cell (four surface atoms per two molecules in the direction of the polymer) together with a 3 × 6 *k*‐point sampling. Transition states of reactions were found using a combination of the climbing image nudge elastic band (CI‐NEB)^[^ ^50^ ^]^ and Dimer^[^ ^51^ ^]^ methods, where CI‐NEB was used to provide an initial guess of a transition state, to be refined by the Dimer method. All structures (local minima as well as transition states) were geometrically optimized until the residual forces on all atoms, except those of the bottom two layers of the slab, were smaller than 0.01 eV Å^−1^. STM simulations were performed for the freestanding networks within the framework of the Tersoff–Hamann approximation^[^ ^52^ ^]^ as implemented by Lorente and Persson.^[^ ^53^ ^]^

Complementary theoretical calculations were performed to simulate the conformation of polymer **P2** in the gas phase, without and with hydrogenation of the nitrogen atoms of the pyrazine moieties, using the FHI‐AIMS package^[^ ^54^ ^]^ and the hybrid exchange–correlation functional B3LYP[47].^[^ ^55^ ^]^ Systems were allowed to relax until the remaining atomic forces reached below 10–2 eV Å^−1^. In all calculations and for all atomic species, the default light basis sets were used.

## Conflict of Interest

The authors declare no conflict of interest.

## Supporting information

Supporting Information

## Data Availability

The data that support the findings of this study are available from the corresponding author upon reasonable request.
