# Supplementary material for: Surface‐Assisted Synthesis of N‐Containing π‐Conjugated Polymers
Source: Adv Sci (Weinh). 2022 May 22;9(19):2200407. doi: 10.1002/advs.202200407 (PMC9259725; doi:10.1002/advs.202200407)
Supplement: Supplementary file 1 — Supporting Information [file ADVS-9-2200407-s001.pdf]

ADVANCED SCIENCE
Open Access

Supporting Information

for *Adv. Sci.*, DOI 10.1002/advs.202200407

Surface-Assisted Synthesis of N-Containing π-Conjugated Polymers

Ana Sánchez-Grande, José I. Urgel, Inés García-Benito, José Santos, Kalyan Biswas, Koen Lauwaet, José M. Gallego, Johanna Rosen, Rodolfo Miranda, Jonas Björk*, Nazario Martín* and David Écija**

Supporting Information

Surface-assisted Synthesis of N-containing π-Conjugated Polymers

Ana Sánchez-Grande, José I. Urgel, Inés Garcia-Benito, José Santos, Kalyan Biswas, Koen Lauwaet, José M. Gallego, Johanna Rosen, Rodolfo Miranda, Jonas Björk,* Nazario Martin,* and David Écija**

A. Sánchez-Grande, J. I. Urgel, I. García-Benito, K. Biswas, K. Lauwaet, R. Miranda, N. Martín and D. Écija
IMDEA Nanoscience, C/ Faraday 9, Campus de Cantoblanco, 28049 Madrid, Spain.
E-mail: jose-ignacio.urgel@imdea.org, nazmar@quim.ucm.es, david.ecija@imdea.org

I. García-Benito, J. Santos and N. Martín
Departamento de Química Orgánica. Facultad de Ciencias Químicas, Universidad Complutense 28040 Madrid, Spain.

J. M. Gallego
Instituto de Ciencia de Materiales de Madrid, CSIC, Cantoblanco, 28049 Madrid, Spain

R. Miranda
Departamento de Física de la Materia Condensada, Universidad Autónoma de Madrid, 28049 Madrid, Spain

J. Rosen and J. Björk
Department of Physics, Chemistry and Biology, IFM, Linköping University 58183 Linköping, Sweden
E-mail: jonas.bjork@liu.se

1. Synthesis

1.1. General methods.

Chemicals and reagents were purchased from commercial suppliers and used as received. Analytical thin-layer chromatography (TLC) was performed using aluminum-coated Merck Kieselgel 60 F254 plates. Purification by column chromatography was performed using silica gel (Merck, Kieselgel 60, 230-240 mesh or Scharlab 60, 230–240 mesh). NMR spectra were recorded on a Bruker Advance 300 (^{1}H: 400 MHz; ^{13}C: 101 MHz) spectrometer at 298 K using partially deuterated solvents as internal standards. Coupling constants (*J*) are denoted in Hz and chemical shifts (δ) in ppm. Multiplicities are denoted as follows: s = singlet, d = doublet, t = triplet, m = multiplet.

1.2. Synthetic details and characterization.

The synthetic route for preparing **4Br4AzaPn** is shown in Scheme S1. Firstly, 5,7,12,14-tetraaza-5,14-dihydro-pentacene (**1**) and 5,7,12,14-tetraaza-6,13-pentacendione (**2**) were prepared according to the literature methods.[1] Then, 15,15,16,16-tetrabromo-5,7,12,14-tetraaza-6,13-pentacene-*p*-quinodimethane (**3, 4Br4AzaPn**) was synthesized according to previously reported synthetic procedures[2] and showed identical spectroscopic properties to those reported therein: ^{1}H NMR (400 MHz, $CDCl_3$): δ (ppm) 8.16 (m, 1H, CH), 7.84 (s, 1H, CH); ^{13}C NMR (101 MHz, $CDCl_3$): δ (ppm) 146.69, 140.59, 134.99, 131.34, 129.51, 101.87.

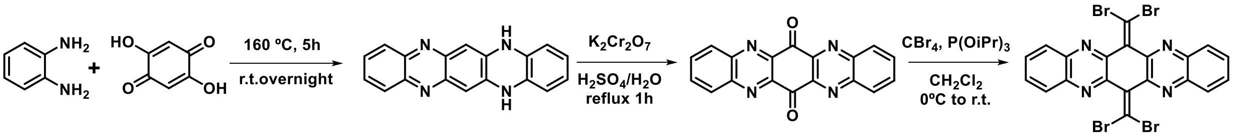

1 2 3

Scheme S1. Synthetic route for the preparation of 15,15,16,16-tetrabromo-5,7,12,14-tetraaza-6,13-pentacene-*p*-quinodimethane (**4Br4AzaPn**).

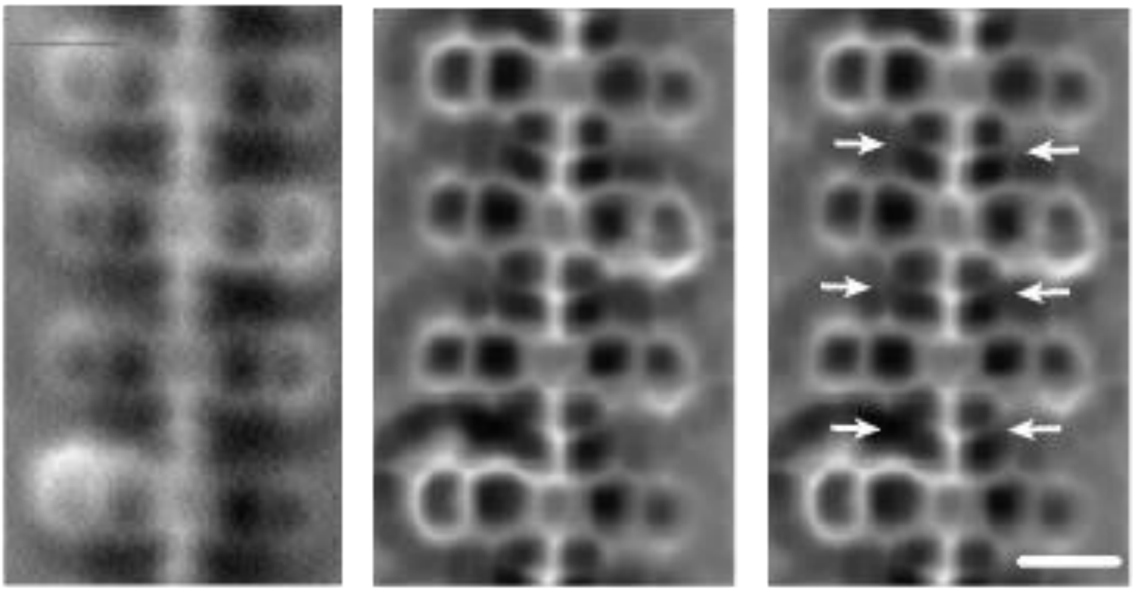

Figure SI1. Nc-AFM image and its corresponding Laplace filtered image of P1 on Ag(111). White arrows point to the positions that correspond to Ag adatoms coordinated to the nitrogen atoms of the tetraazapentacene units (V_b = 3 mV and scale bar = 6 Å).

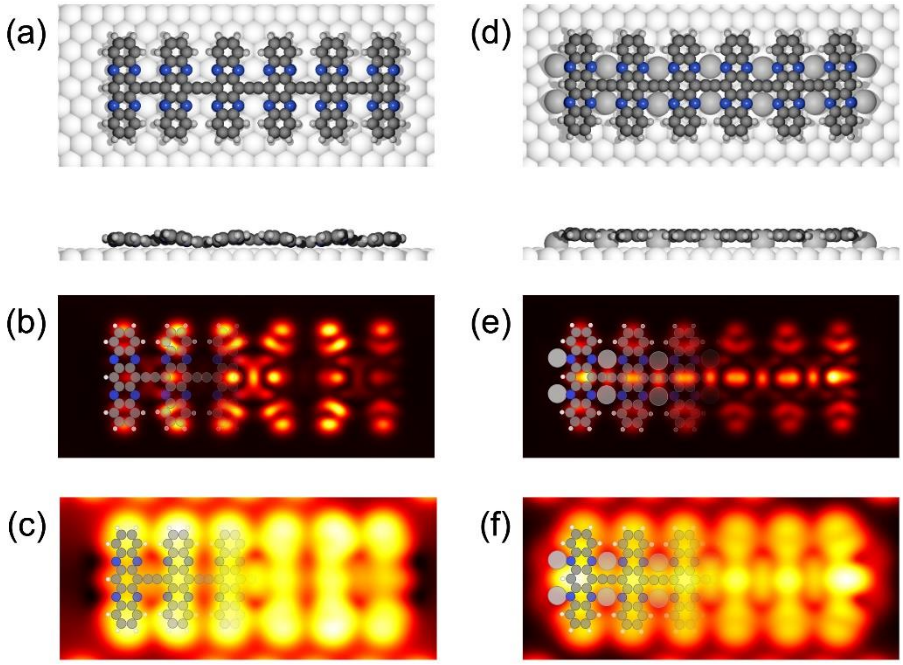

Figure SI2. Comparison of simulated STM images around the Fermi level of P1 without and with silver adatoms. a-c) The **P1** oligomer consisting of six monomer units on the flat Ag(111) surface showing (a) the most stable adsorption geometry and (b) the corresponding local density of states (LDOS) at constant height at the Fermi level ($\boldsymbol{E_F}$) and (c) the constant integrated LDOS between $\boldsymbol{E_F}$ and $\boldsymbol{E_F - 1.0}$ eV. d-f) The **P1** oligomer consisting of six monomer units with the nitrogen atoms coordinated to Ag adatoms on the Ag(111) surface showing (d) the most stable adsorption geometry and (e) the corresponding local density of states (LDOS) at constant height at the Fermi level ($\boldsymbol{E_F}$) and (f) the constant integrated LDOS between $\boldsymbol{E_F}$ and $\boldsymbol{E_F - 1.0}$ eV. The simulated images at constant height and constant LDOS corresponds to experimental images at low bias voltage obtained at in the constant height and constant current mode, respectively.

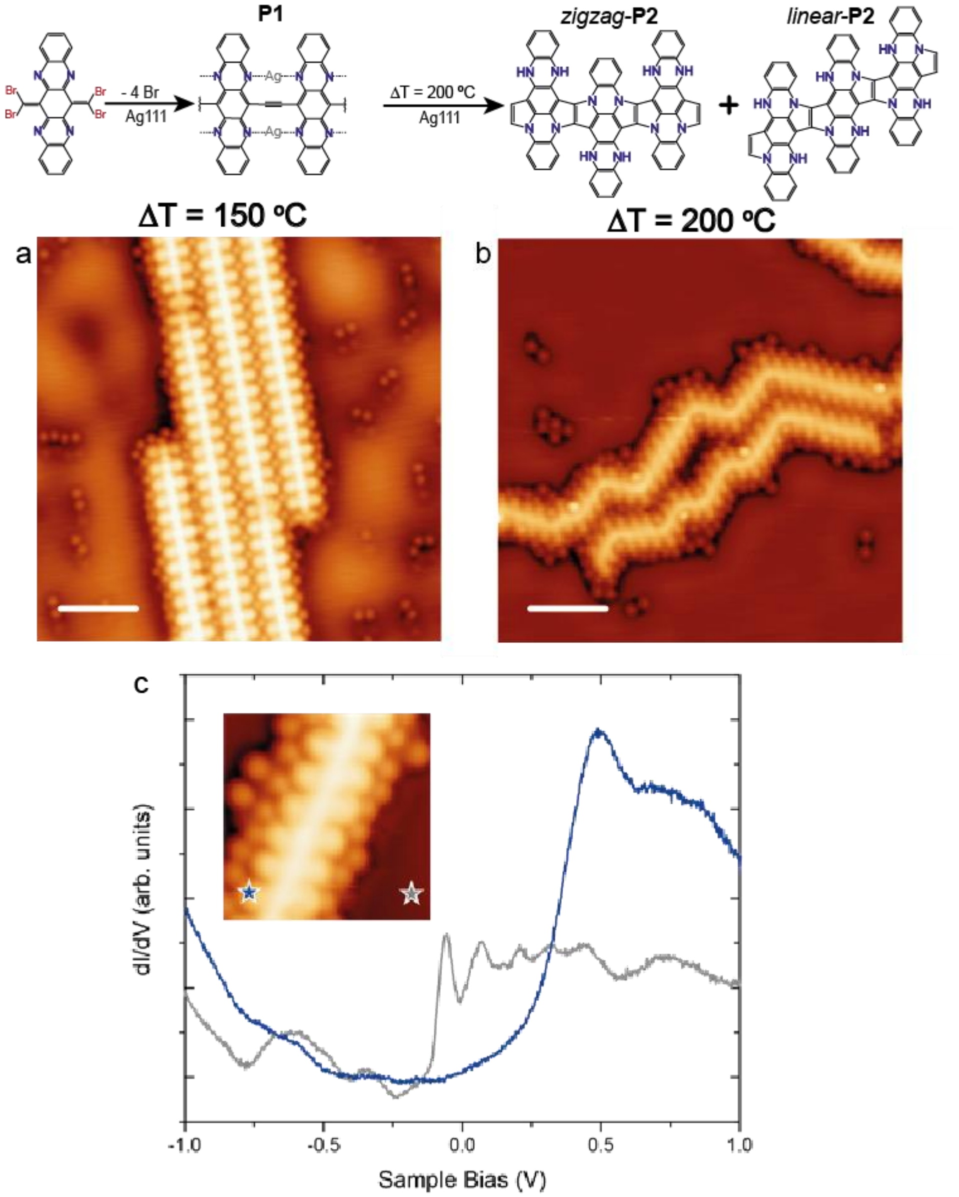

Figure SI3. STM overview images of N-doped pentacene-based precursor on Ag(111) after thermal activation at distinct temperatures. a) 150 ºC (V_b = 20 mV, I_t = 30 pA and scale bar = 3 nm); b) 200 ºC (V_b = 20 mV, I_t = 20 pA and scale bar = 3 nm). c) Scanning tunneling spectroscopy of **P2** on Ag(111).

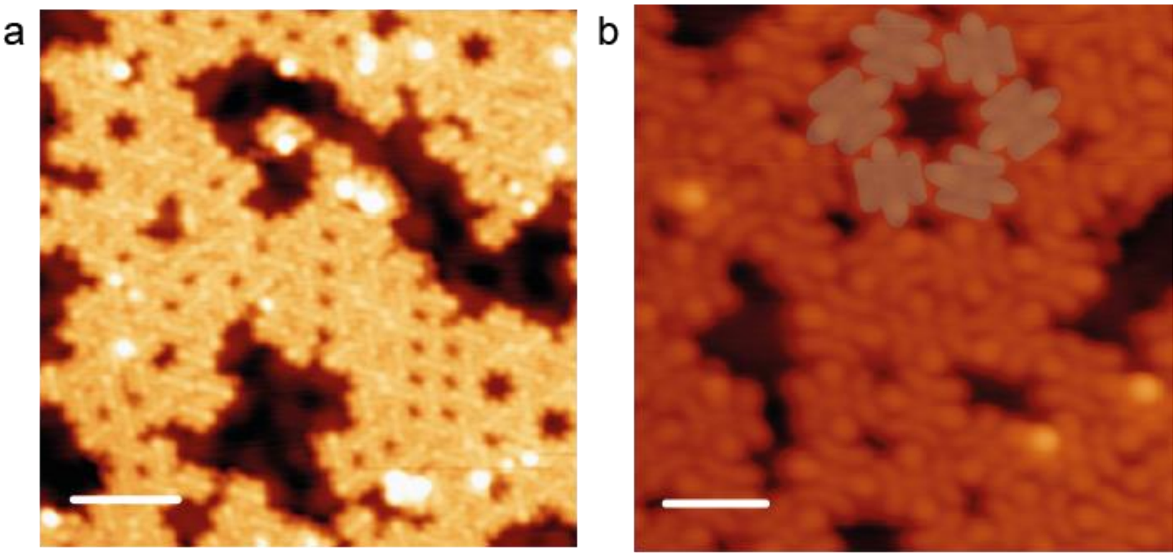

Figure SI4. STM overview images of N-doped pentacene-based precursor on Au(111) with the substrate held at RT. a) V_b = 200 mV, I_t = 100 pA and scale bar = 4 nm. b) V_b = 200 mV, I_t = 100 pA and scale bar = 2 nm. Six individual molecules are highlighted.

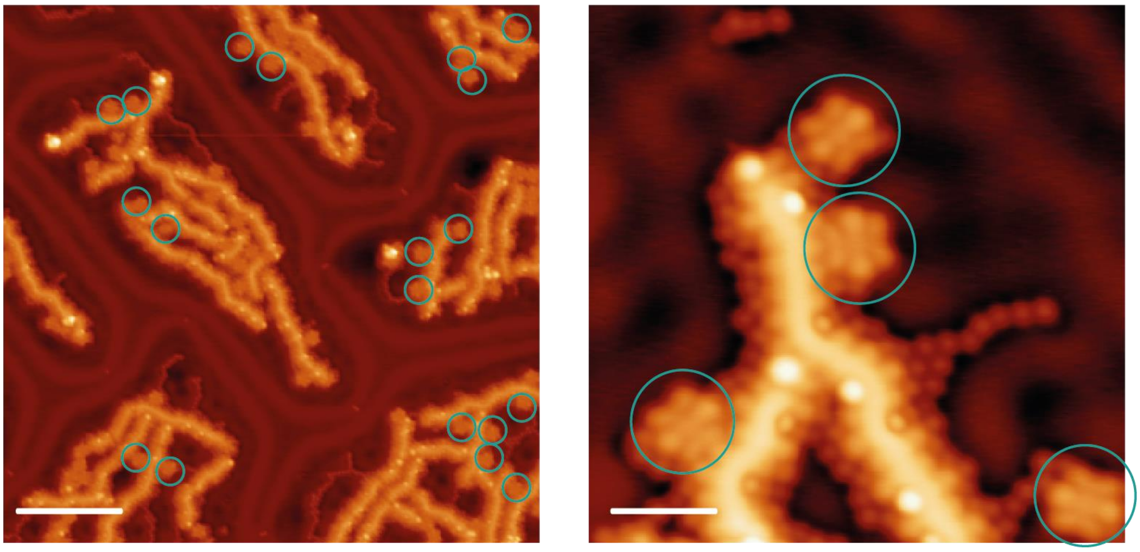

Figure SI5. STM images displaying the coexistence of unreacted 4Br4AzaPn molecules with P2 polymer on Au111 after annealing the substrate at 100 ºC. a) V_b = 200 mV, I_t = 40 pA and scale bar = 10 nm. b) V_b = 20 mV, I_t = 100 pA and scale bar = 2 nm. Unreacted species are highlighted by green circles.

a

b

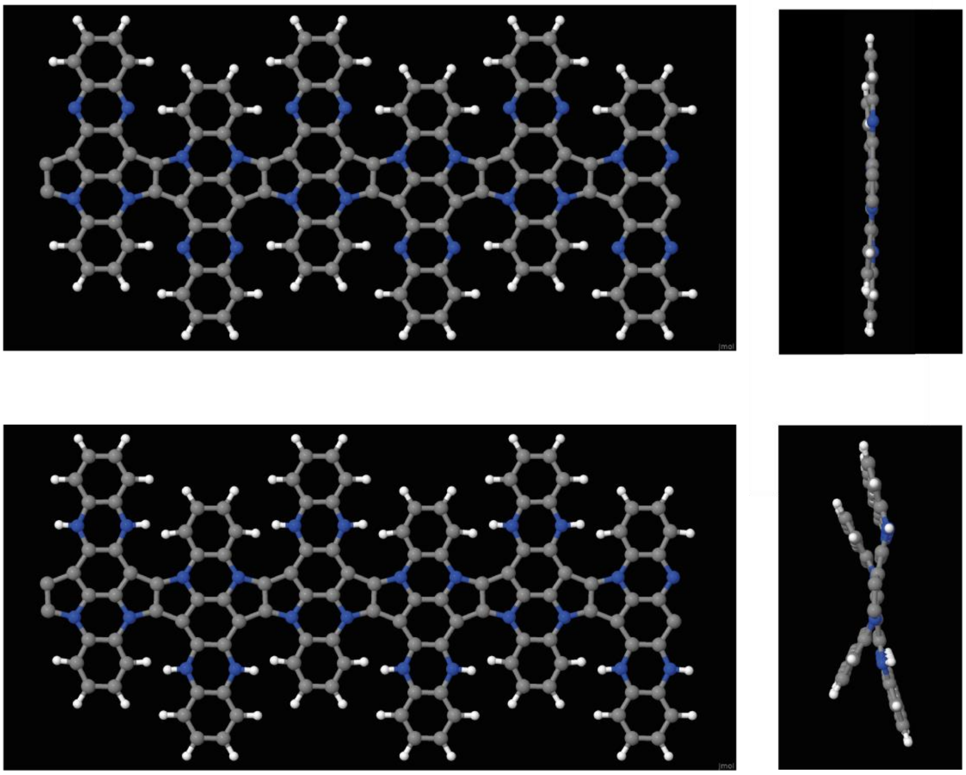

Figure SI6. DFT simulations of the geometry of freestanding polymer P2. a) Without and b) with hydrogenation of nitrogen atoms in pyrazine moieties. Courtesy of S. Edalatmanesh and P. Jelínek from the Institute of Physics (Czech Academy of Sciences).

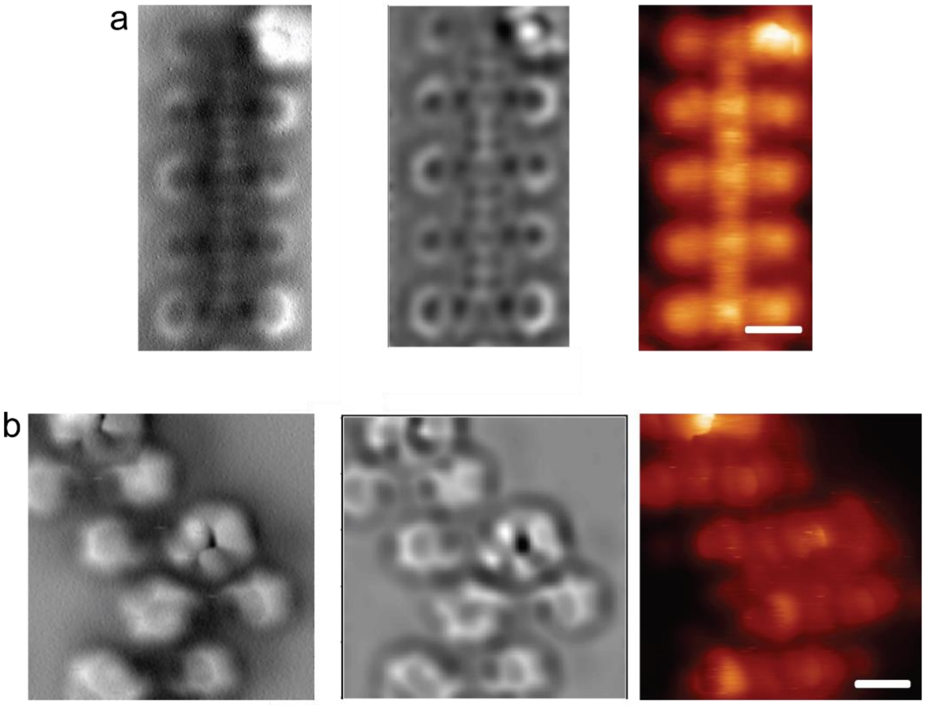

Figure SI7. Formation of P1 polymers and P3 monomers by the deposition of 4Br4AzaPn species on Ag(100) at room temperature. Nc-AFM images (left panels), their corresponding Laplace filtered STM images (middle panels) and constant-height STM images (right panels) of **P1** (top panels) and **P3** (bottom panels) on Ag(100). a-b) V_b = 3 mV and scale bar = 0.5 nm.

DFT calculations of P3 on Ag(100)

To model the structure of P3 species, we first calculated different adsorption configurations of a single molecule with the $=CBr_2$ groups removed, both with and without hydrogen atoms bonded to the central carbon atoms of the molecule. The adsorption configurations, with their relative energies, are shown in Figure SI8 and SI9 without and with hydrogen terminations, respectively.

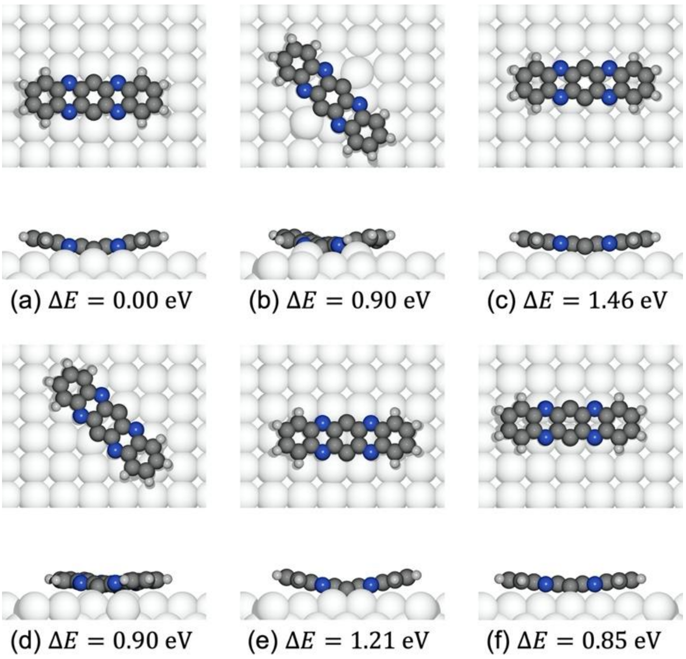

Figure SI8. Adsorption configurations of a monomer on Ag(100) with the $=CBr_2$ groups removed. Relative energies are indicated with respect to the most stable adsorption configuration (a).

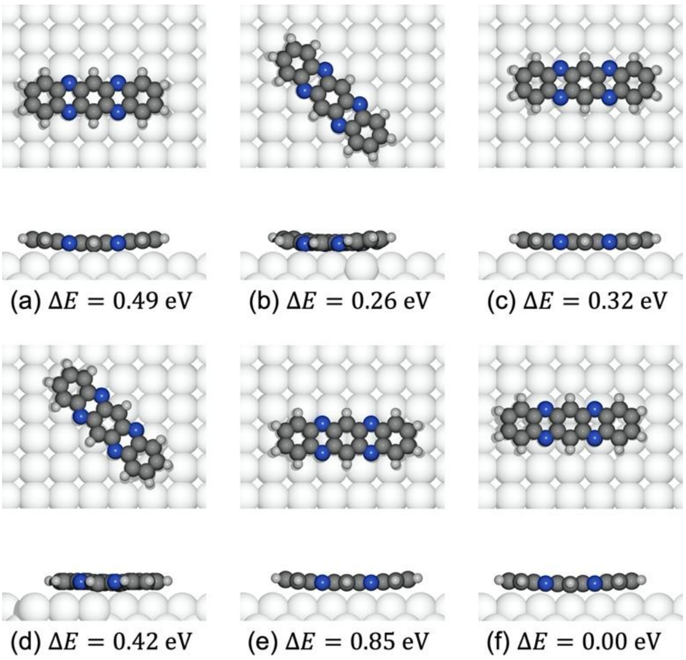

Figure. SI9. Adsorption configurations of a **P3** monomer on Ag(100) with the $=CBr_2$ groups replaced by hydrogen atoms. Relative energies are indicated with respect to the most stable adsorption configuration (f).

Based on the most stable adsorption configurations both with and without H on the former $=CBr_2$ sites, periodic self-assembled structures were constructed and geometrically optimized. For the optimized geometries we performed STM simulations. The optimized structures are shown in Figure SI10 and the corresponding STM simulations in Figure SI11.

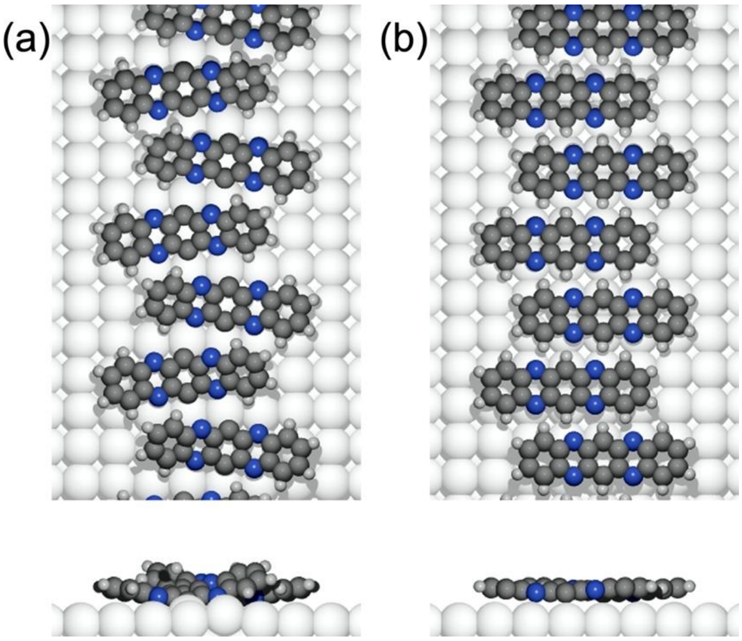

Figure SI10. P3 species on Ag(100) with the $=CBr_2$ moieties from the **4Br4AzaPn** precursor (a) removed and (b) replaced by hydrogen atoms. The two structures were obtained based on the most stable adsorption configurations in Figure SI8a and SI9f, respectively.

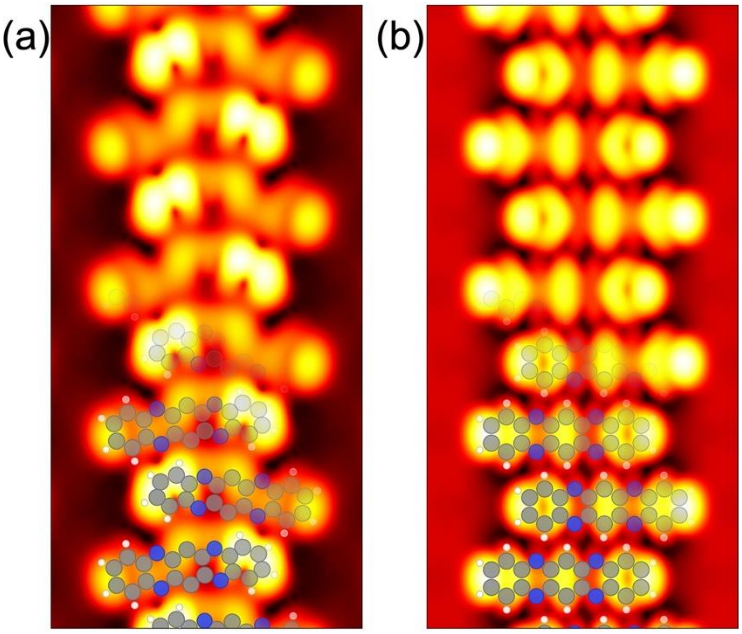

Figure SI11. STM simulations of the two types of **P3** species on Ag(100) with the $=CBr_2$ moieties from the **4Br4AzaPn** precursor (a) removed and (b) replaced by hydrogen atoms. The STM simulations were obtained from the local density states (LDOS) around the Fermi, plotted for constant LDOS. This corresponds to constant current images obtained for small bias voltages.

Coupling mechanisms

For the calculations of coupling mechanisms on Ag(111) and Au(111) we started from dehalogenated two molecules in the most stable adsorption configurations on the two surfaces. In other words, we first investigated the most stable adsorption configurations on the two surfaces. In Figure SI12 and SI13 the most stable adsorption configurations of the dehalogenated molecules are shown for Ag(111) and Au(111), respectively, and the coupling mechanisms are shown in Figure SI14 and SI15.

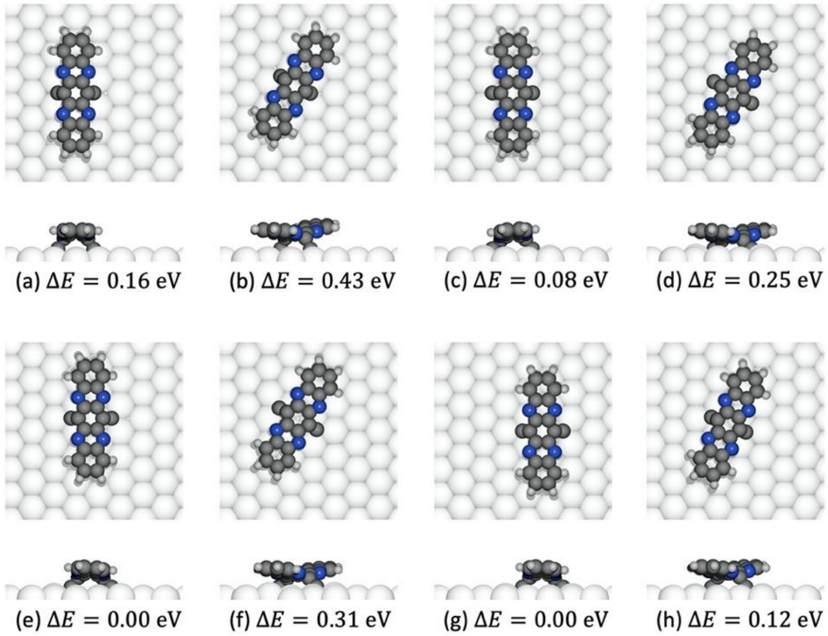

Figure SI12. Adsorption configuration of dehalogenated monomer on Ag(111). For each configuration the energy is given with respect to the most stable configuration (e).

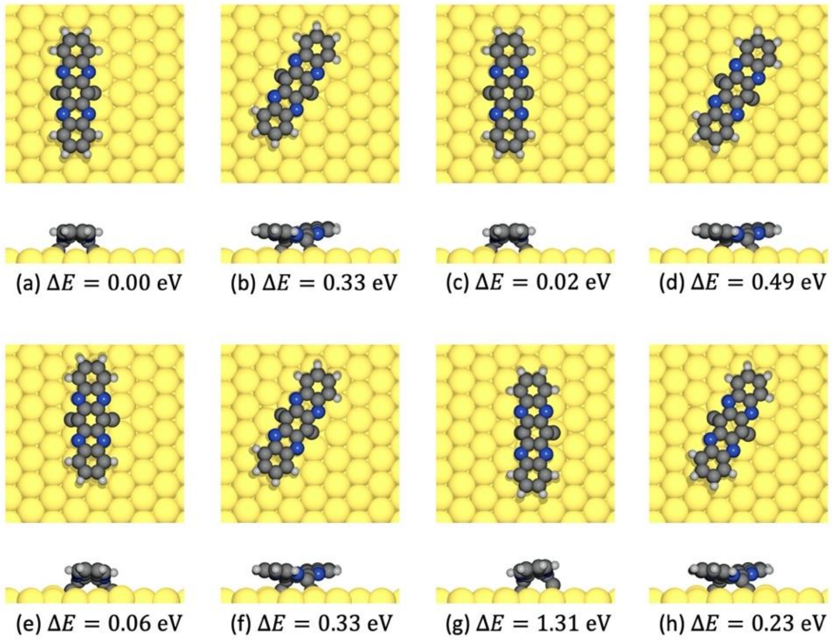

Figure SI13. Adsorption configuration of dehalogenated monomer on Au(111). For each configuration the energy is given with respect to the most stable configuration (e).

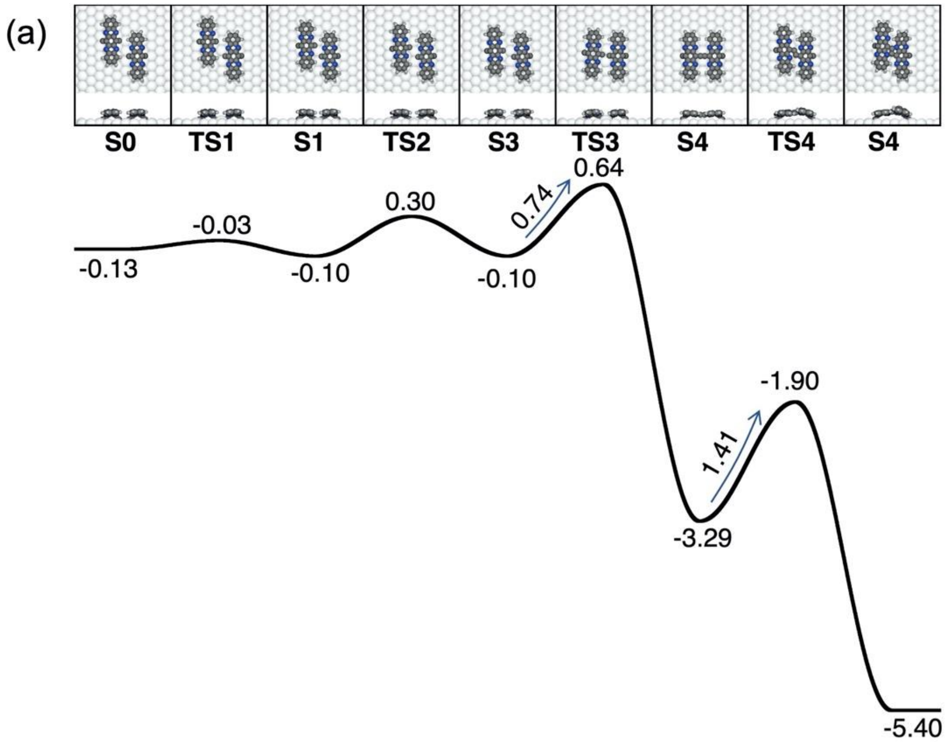

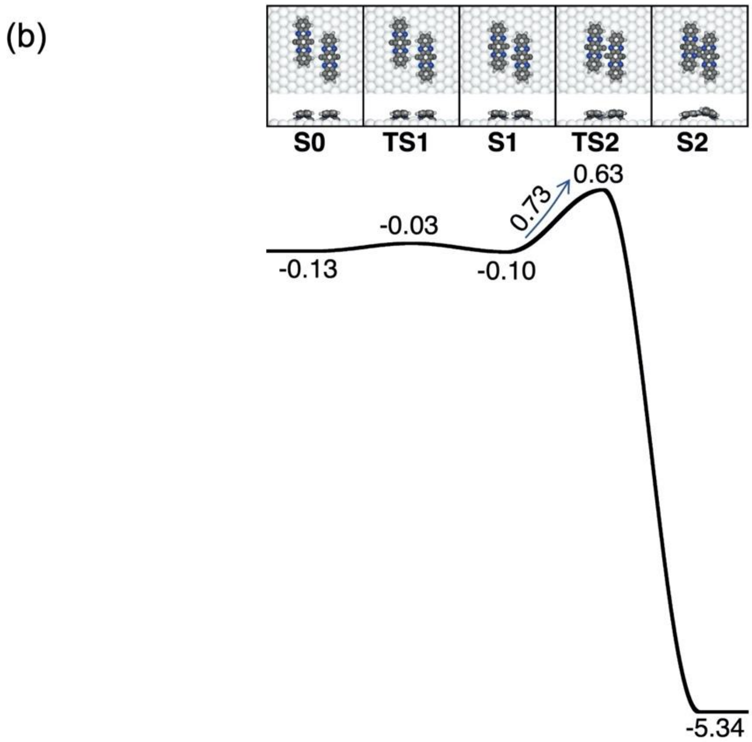

Figure SI14. Computed reaction mechanisms for the coupling between two dehalogenated monomers on the Ag(111) surface. (a) The stepwise formation of an ethenylene bridge and its subsequent transformation into a pyrrole-pyrrole group and (b) the direct formation of a pyrrole-pyrrole group, including top and side views of local minima (**S0**, **S1**, …) and transition states (**TS1**, **TS2**, …) and corresponding energy profile. The energies are given with respect to two dehalogenated molecules separated from each other on the surface. These energies were used to construct the simplified energy diagrams for the coupling on Ag(111) shown in Figure 4a of the main manuscript. Units in eV.

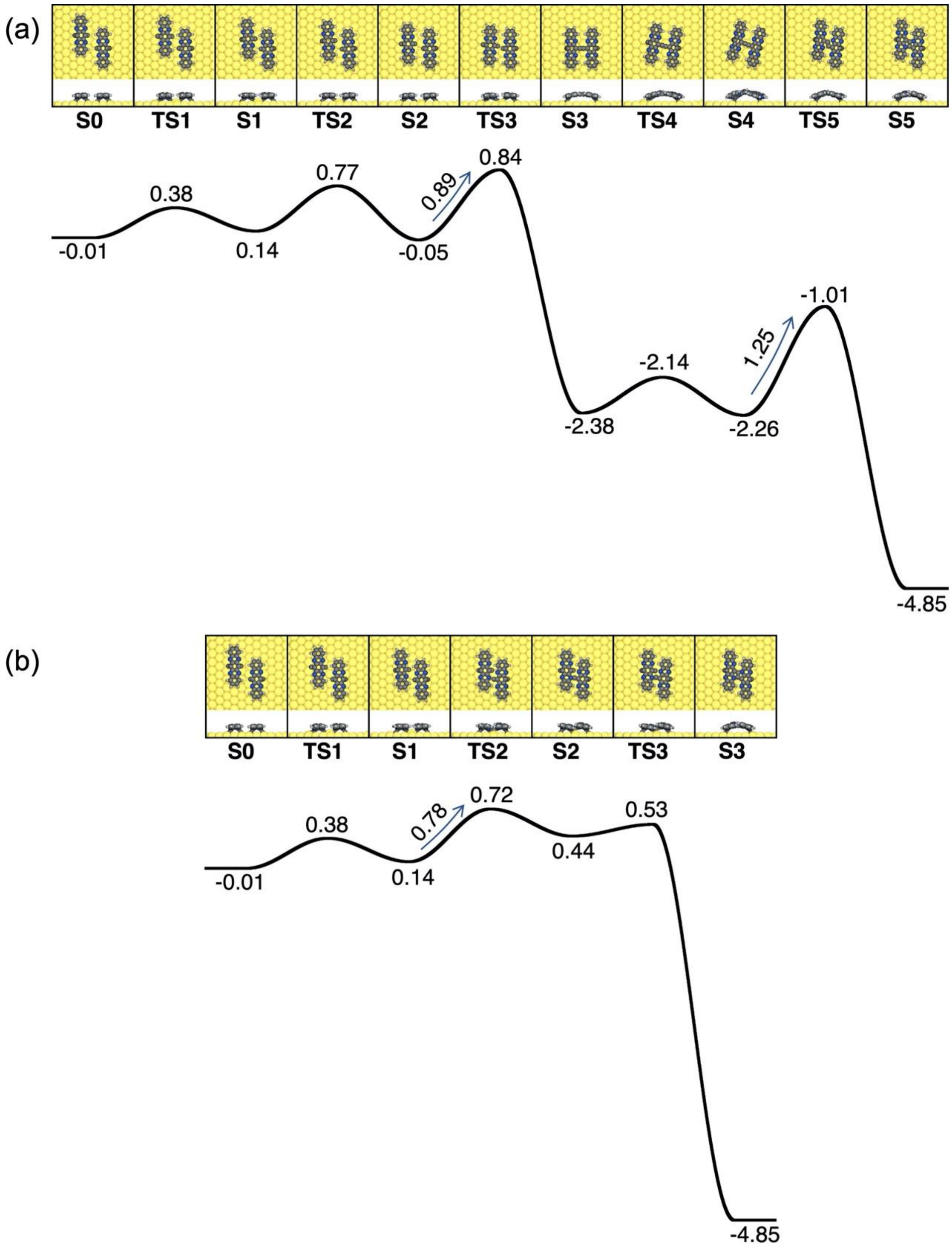

Figure SI15. Computed reaction mechanisms for the coupling between two dehalogenated monomers on the Au(111) surface. showing (a) the stepwise formation of an ethenylene bridge and its subsequent transformation into a pyrrole-pyrrole group and (b) the direct formation of a pyrrole-pyrrole group, including top and side views of local minima (**S0**, **S1**, …) and transition states (**TS1**, **TS2**, …) and corresponding energy profile. The energies are given with respect to two dehalogenated molecules separated from each other on the surface. These energies were used to construct the simplified energy diagrams for the coupling on Au(111) shown in Figure 4a of the main manuscript. Units in eV.

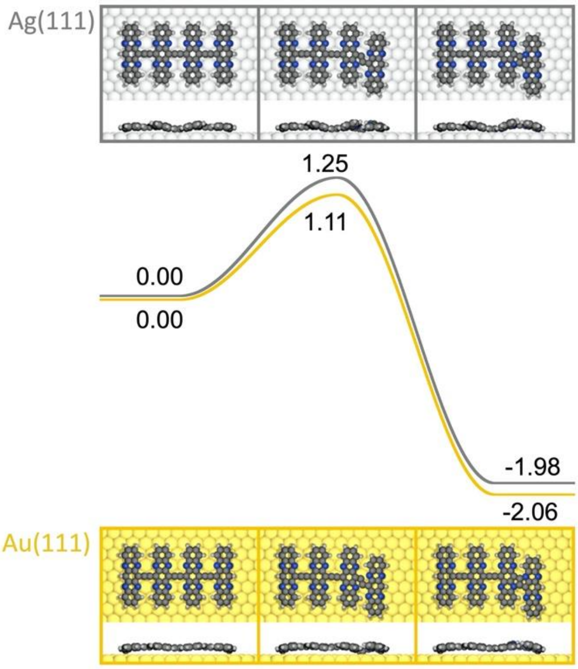

Figure SI16. Reaction of oligomer on surfaces. Computed reaction mechanisms for the transformation of an ethynylene bridge into a pyrrole-pyrrole groups for an ethynylene-coupled oligomer consisting of four units on the flat Ag(111) and Au(111), showing top and side views of initial, transition state and final state for the reactions on the two surface as well as corresponding energy profiles, as indicated. Units in eV.

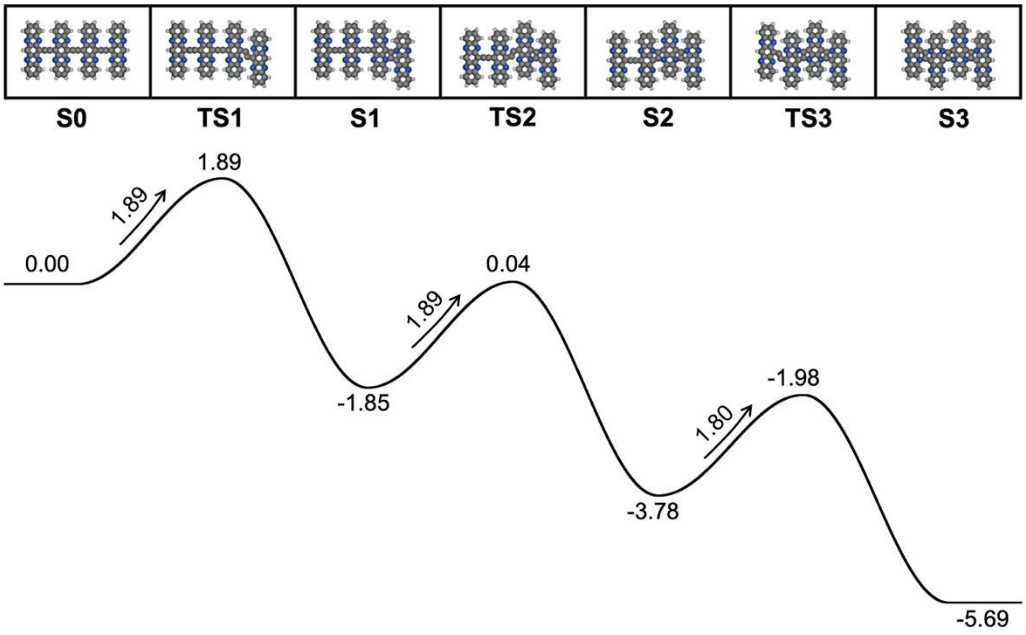

Figure SI17. Reactions of oligomer in gas phase. Computed reaction mechanism for the step-wise transformation of a four-unit oligomer connected by ethynylene bridges (**S0**) to an oligomer with pyrrole-pyrrole connects (**S3**) in gas-phase, depicting local minima (**S0**, **S1**, …) and transition states (**TS1**, **TS2**, …) and corresponding energy profile. Units in eV.

Adsorption configurations of oligomers on Au(111) and Ag(111)

For the calculations of the oligomer transformation reactions different adsorption configurations were considered for both reactants and products, shown in Figure SI18-SI21 for the atomically flat surfaces. Furthermore, on Ag(111) we considered the reaction with Ag adatoms, for which different possible initial configurations are shown in Figure SI22 (the most stable one was used as starting point for the reaction shown in Figure 4b of the main manuscript.) Finally, on Ag(111) we also did calculations of an oligomer consisting of six units, both without and with adatoms (Figure SI23-SI24). The most stable configuration in each case was used for the STM simulations.

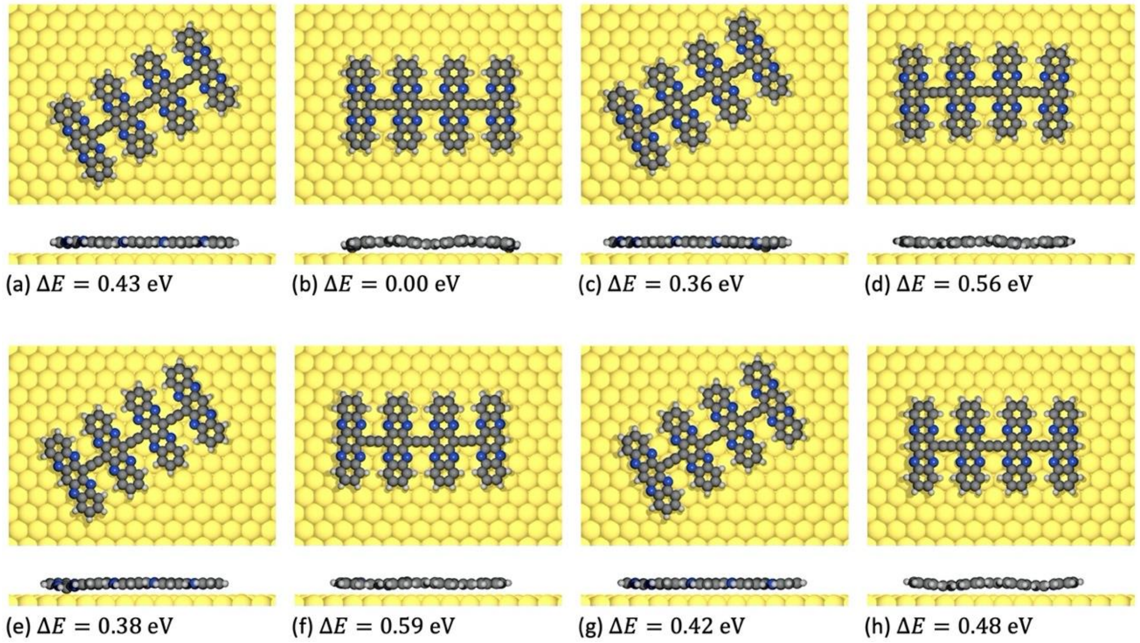

Figure SI18. Adsorption configurations on Au(111). The oligomer consisting of four monomers connected by ethynylene bonds on the Au(111) surface. For each configuration, the energy is given with respect to the most stable adsorption site (b).

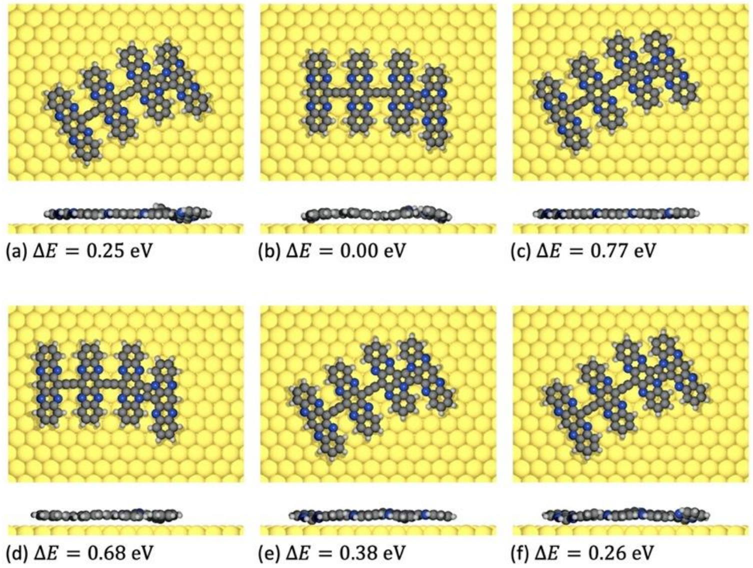

Figure SI19. Adsorption configurations on Au(111). The oligomer consisting of four monomers, with one of the etheylene bonds transformed to a pyrrole-pyrrole group, on the Au(111) surface. For each configuration, the energy is given with respect to the most stable adsorption site (b).

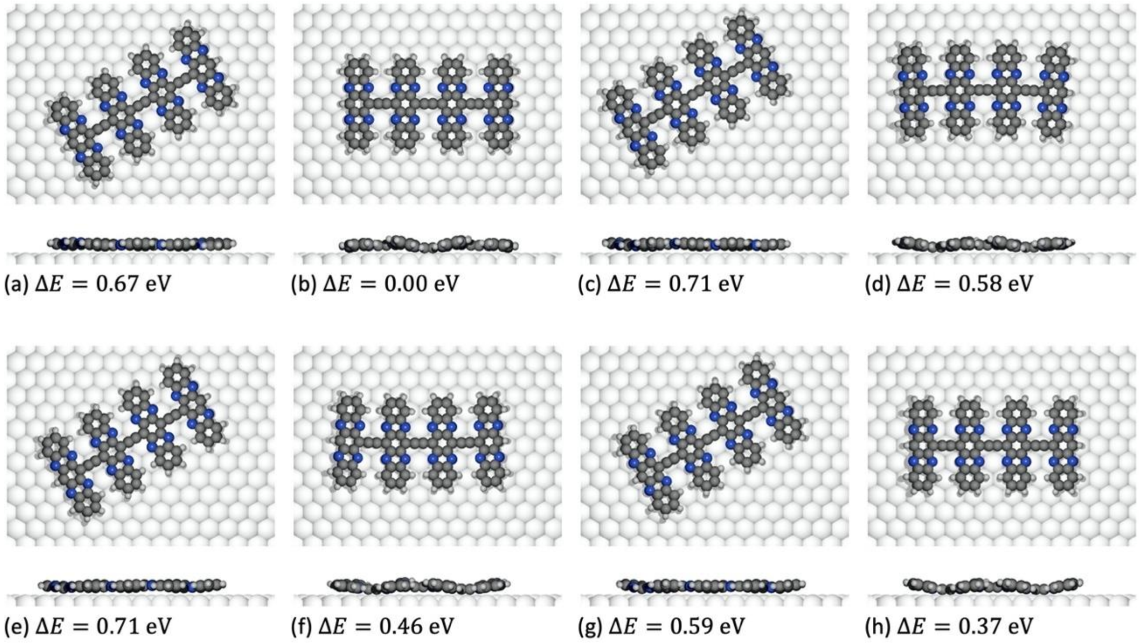

Figure SI20. Adsorption configurations on Ag(111). The oligomer consisting of four monomers connected by ethynylene bonds on the Ag(111) surface. For each configuration, the energy is given with respect to the most stable adsorption site (b).

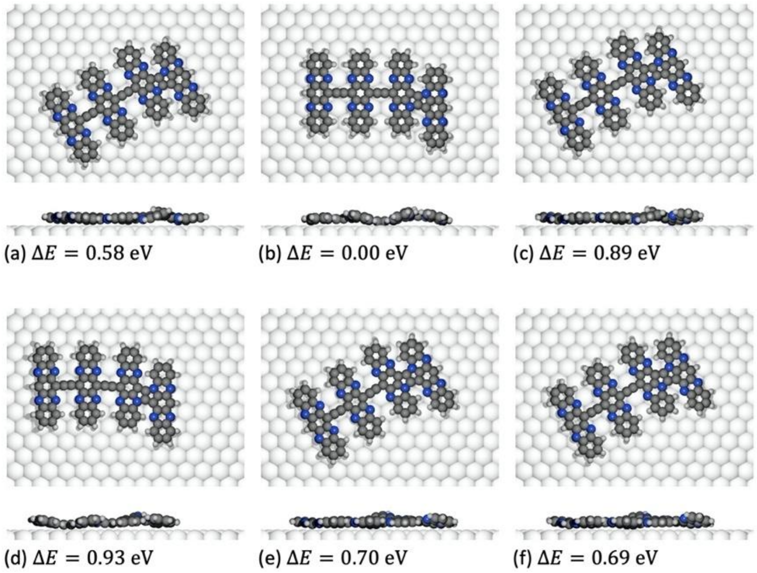

Figure SI21. Adsorption configurations on Ag(111). The oligomer consisting of four monomers, with one of the etheylene bonds transformed to a pyrrole-pyrrole group, on the Ag(111) surface. For each configuration, the energy is given with respect to the most stable adsorption site (b).

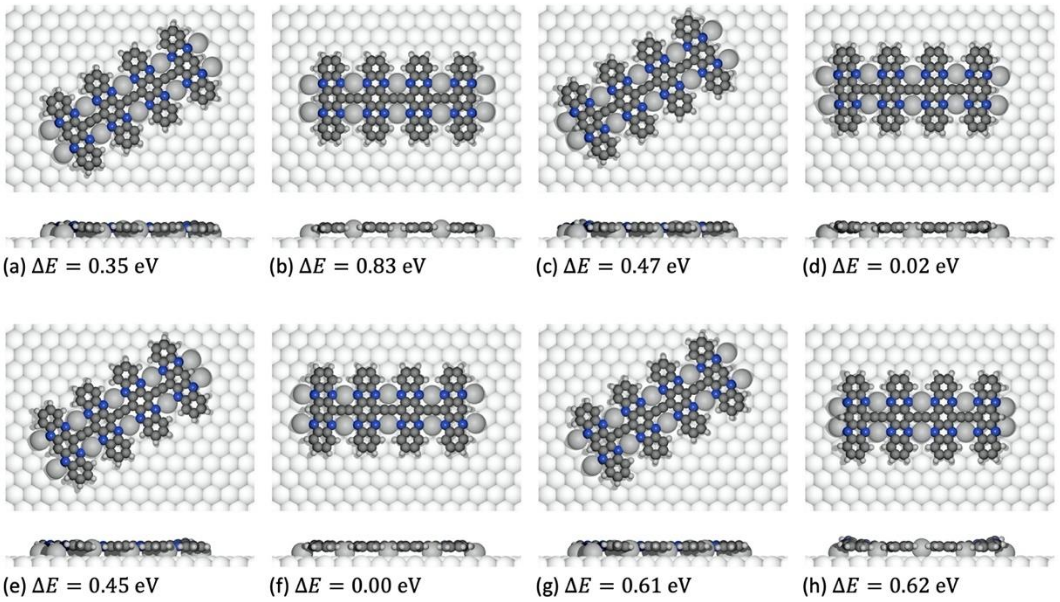

Figure SI22. Adsorption configurations on Ag(111) with adatoms. Adsorption configurations considered for the oligomer consisting of four monomers connected by ethynylene bonds on the Ag(111) surface with the nitrogen atoms coordinated to Ag adatoms (rendered darker than surface atoms). For each configuration, the energy is given with respect to the most stable adsorption site (f).

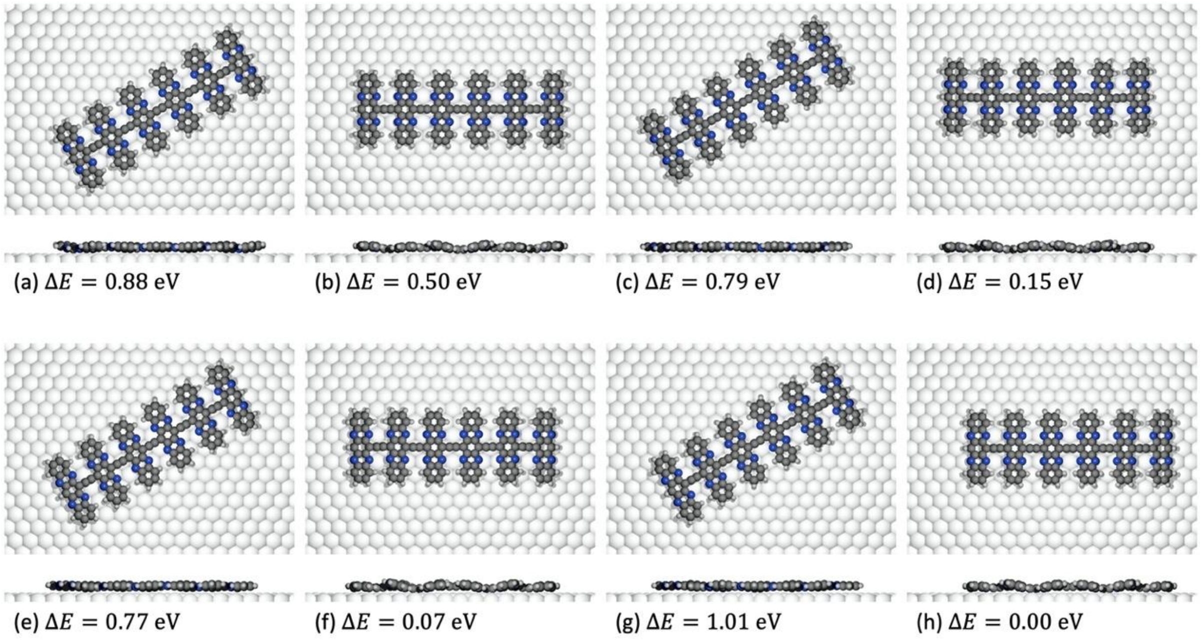

Figure SI23. Adsorption configurations on Ag(111). Adsorption configurations considered for the oligomer consisting of six monomers connected by ethynylene bonds on the Ag(111) surface. For each configuration, the energy is given with respect to the most stable adsorption site (h).

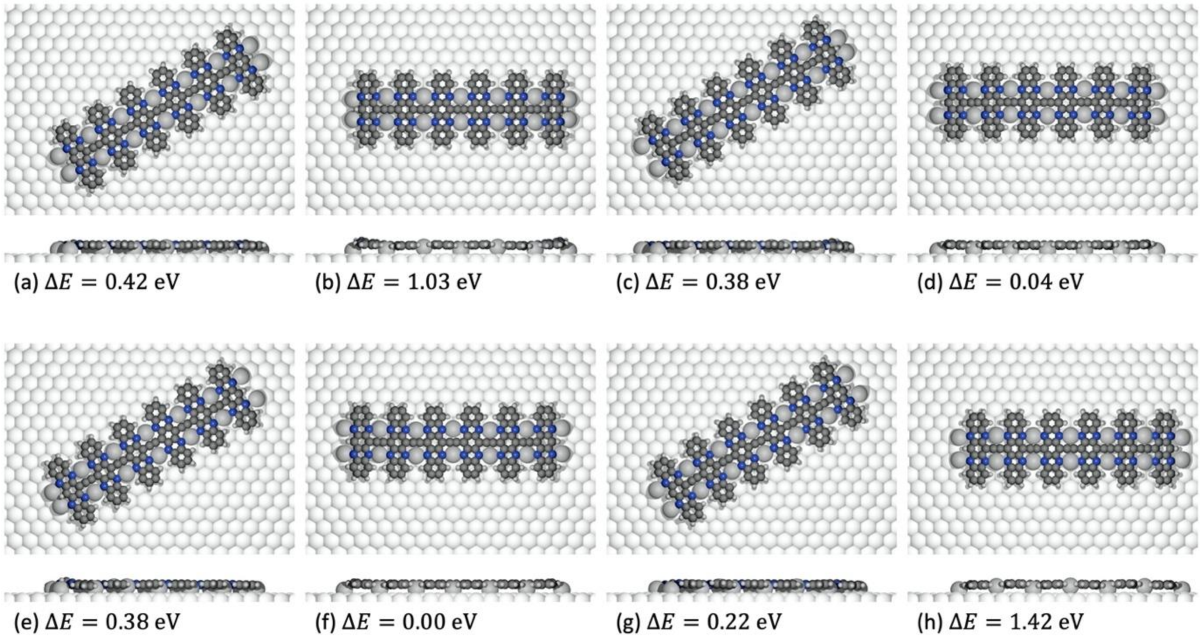

Figure SI24. Adsorption configurations on Ag(111) with adatoms. Adsorption configurations considered for the oligomer consisting of six monomers connected by ethynylene bonds on the Ag(111) surface with the nitrogen atoms coordinated to Ag adatoms (rendered darker than surface atoms). For each configuration, the energy is given with respect to the most stable adsorption site (f).

Adatom entropy considerations

To estimate the entropy of Ag adatoms on the Ag(111) surface we employed the complete potential energy sampling (CPES)[3]. In this approach, the translational partition function at a temperature T is given by

$$Z_{\mathrm{T}}^{\mathrm{CPES}} = \frac{A}{A_{\mathrm{cell}}} \frac{2\pi m k_{\mathrm{B}} T}{h^2} \iint \exp\left(\frac{-V(x,y)}{k_B T}\right) dx\, dy,$$

where m is the mass (of the Ag adatom in this case), k_{B} is Boltzmann's constant and h is Planck's constant. $V(x,y)$ is the potential energy of the adatom at different lateral positions x and y. The integration is performed numerically over a surface cell of area A_{cell} while the area A is the available area per adatom (i.e., the inverse of the adatom surface concentration). The fraction $\frac{A}{A_{\mathrm{cell}}}$ is needed to include the concentration of adatoms into the partition function. If the adatoms are assumed to move freely on the surface, in other words $V(x,y) = 0$, the partition function reduces to the free translator (FT) partition function $Z_{\mathrm{T}}^{\mathrm{FT}}$, *i.e.*, the translational partition function for a gas of one particle confined to an area A

$$Z_{\mathrm{T}}^{\mathrm{FT}} = A \frac{2\pi m k_{\mathrm{B}} T}{h^2}.$$

Using either of the partition functions, the entropy in the canonical ensemble is given according to

$$S = -\frac{\partial}{\partial T}(-k_B T \ln Z)_{V,N} = k_B \ln Z + k_B T \left(\frac{\partial \ln Z}{\partial T}\right)_{V,N}.$$

For the free-translator partition function, this gives the 2D Sackur-Tetrode equation.

To calculate the potential energy landscape $V(x,y)$ used to calculate $Z_{\mathrm{T}}^{\mathrm{CPES}}$ we mapped the potential energy surface of an Ag adatom on the Ag(111) surface by freezing the lateral position of the Ag adatom at positions on the surface while allowing optimization of remaining degrees of freedom (including the uppermost layers of the Ag slab). The resulting potential energy surface is shown in Figure SI25. This potential energy surface was then used to construct the translational entropy within the CPES picture, and compared to the free-translator model, for different surface coverages and temperatures in Figure SI26. If assuming a surface concentration of 0.1 nm^{-2} (close to the value used in the article mentioned by the reviewer) the entropy is 0.99 meV/K at 200 ℃ according to the CPES calculations, which corresponds to a free energy contribution of -0.47 eV per adatom. Figure SI27 compare the energy profiles with and without the adatom entropy for the ladderization on Ag(111), demonstrating that including adatom entropy lowers the energy profile, making the overall reaction exergonic.

While we would not like to make claims about the precise entropy of the adatoms, it is clear that it does affect the energy landscape and can explain the ladderization at elevated temperatures.

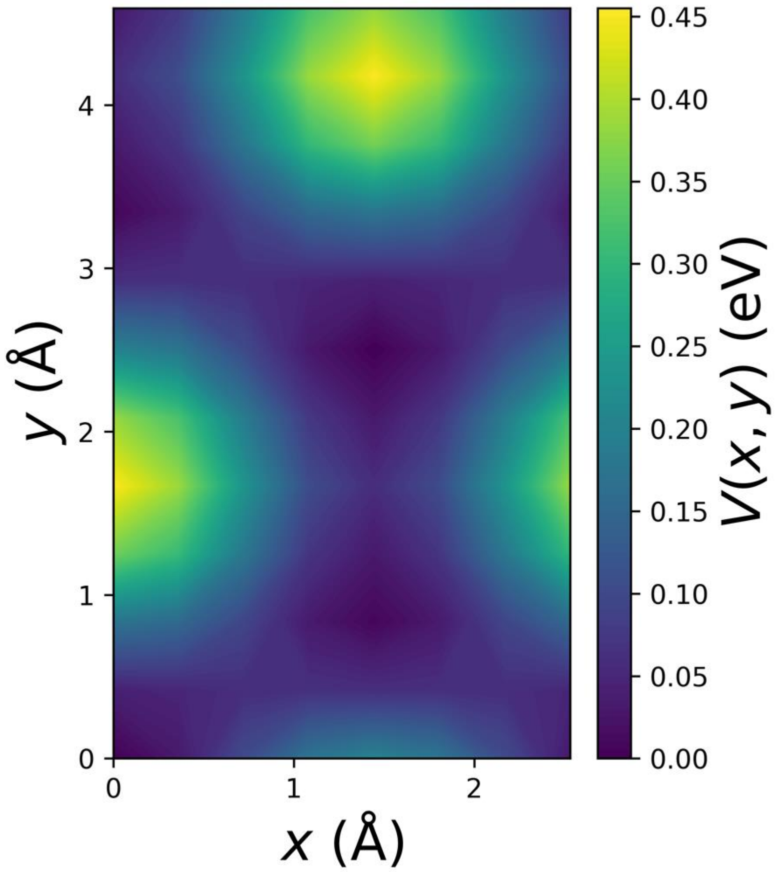

Figure SI25. Potential energy surface (PES) of an Ag adatom on the Ag(111) surface. The PES was calculated by freezing the lateral position of the Ag adatom at positions on the surface while allowing optimization of remaining degrees of freedom (including the two uppermost layers of the Ag slab).

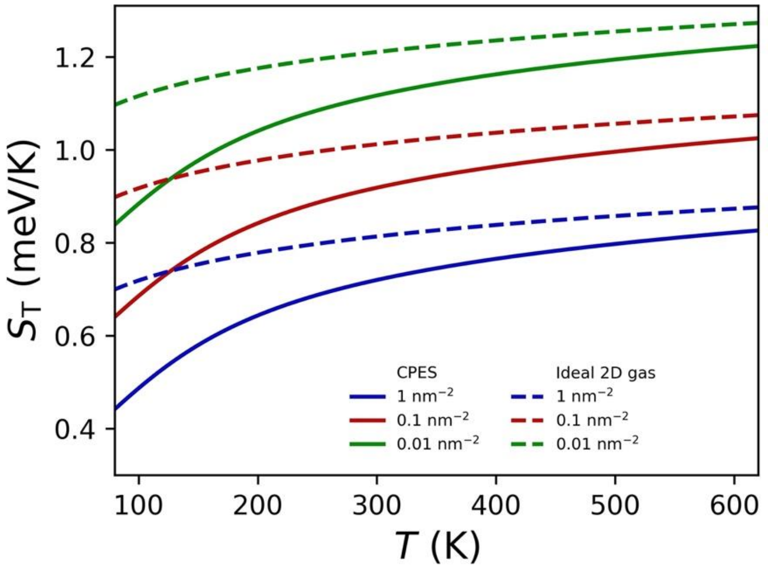

Figure SI26. Calculated translational entropies of Ag adatoms as a function of temperature, comparing the results for an ideal 2D gas and for a complete potential energy sampling (CPES) for different surface coverages, as indicated.

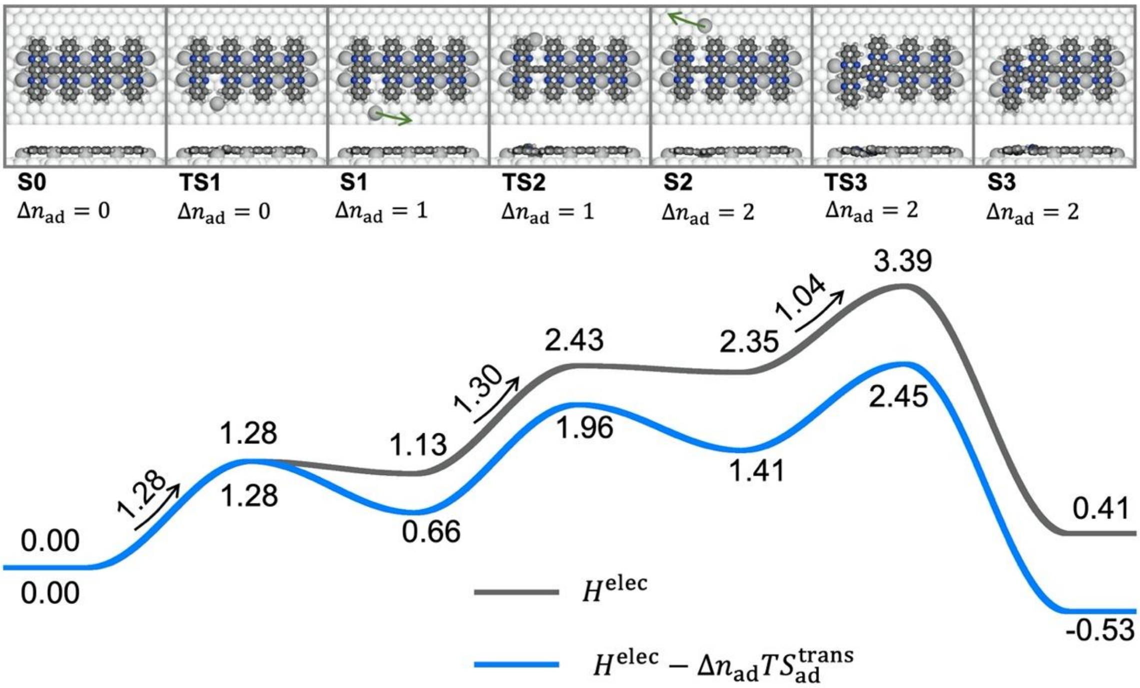

Figure SI27. The reaction mechanism on Ag(111) of removing adatoms (**S0** to **S2**) and the concomitant ladderization of an ethynylene bridge into a pyrrolopyrrole group (**S2** to **S3**), showing local minima (**S0**-**S3**) and transition states (**TS1**-**TS3**) of the pathway. For each state, the quantity Δn_{ad} indicates the number of adatoms removed from the structure. The associated energy profiles were calculated by only considering the electronic enthalphy (H^{elec}) and by including the translational entropy ($S_{\mathrm{ad}}^{\mathrm{trans}}$) of the removed adatoms. The entropy was evaluated at a temperature of 200 ℃ and a surface concentration of adatoms of 0.1 nm^{-2} using a complete potential energy sampling. In Figure SI26 the translational entropy is plotted for different temperatures and surface concentrations. Units in eV.

DFT calculations of the role of Au adatoms

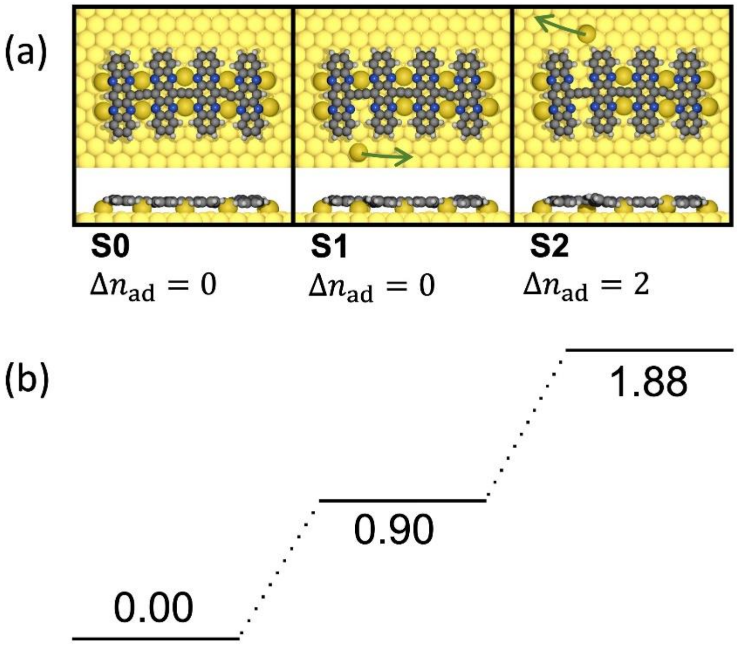

Figure SI28. Calculated results for a hypothetical **P1** oligomer consisting of four molecular units on Au(111) with the nitrogen atoms coordinated to Au adatoms (**S0**), demonstrating the energy cost of removing one (**S1**) and two (**S2**) adatoms from the structure. In (a) the top and side views of the different structures are shown, while the energies of the respective local energy minima with respect to **S0** are displayed in (b) in units of eV. The energies could be compared to the corresponding states in Figure 4 for Ag(111). On Au(111) it requires 0.90 eV to remove one adatom and 1.88 eV to remove two adatoms, while the corresponding numbers for Ag(111) are 1.13 eV and 2.35 eV, respectively. I.e. adatoms bind weaker in the **P1** polymer for Au than Ag, which could be a contributing factor why the **P1** polymer is not observed on Au(111).

References

[1] S. Miao, A. L. Appleton, N. Berger, S. Barlow, S. R. Marder, K. I. Hardcastle, U. H. F. Bunz, *Chem. – Eur. J.* **2009**, *15*, 4990–4993.
[2] Y. Min, C. Dou, H. Tian, J. Liu, L. Wang, *Chem. Commun.* **2019**, *55*, 3638–3641.
[3] M. Jørgensen*, H. Grönbeck*. *J. Phys. Chem. C* **2017**, 121, 7199-7207.
